# Effects of wearing different face masks on cardiopulmonary performance at rest and exercise in a partially double-blinded randomized cross-over study

**DOI:** 10.1038/s41598-023-32180-9

**Published:** 2023-04-28

**Authors:** Eike-Maximillian Marek, Vera van Kampen, Birger Jettkant, Benjamin Kendzia, Bianca Strauß, Kirsten Sucker, Melanie Ulbrich, Anja Deckert, Hans Berresheim, Christian Eisenhawer, Frank Hoffmeyer, Simon Weidhaas, Thomas Behrens, Thomas Brüning, Jürgen Bünger

**Affiliations:** Institute for Prevention and Occupational Medicine of the German Social Accident Insurance, Institute of the Ruhr-University Bochum (IPA), Bürkle‑de‑la‑Camp Platz 1, 44789 Bochum, Germany

**Keywords:** Respiration, Physiology, Cardiology, Health occupations

## Abstract

The use of face masks became mandatory during SARS-CoV-2 pandemic. Wearing masks may lead to complaints about laboured breathing and stress. The influence of different masks on cardiopulmonary performance was investigated in a partially double-blinded randomized cross-over design. Forty subjects (19–65 years) underwent body plethysmography, ergometry, cardiopulmonary exercise test and a 4-h wearing period without a mask, with a surgical mask (SM), a community mask (CM), and an FFP2 respirator (FFP2). Cardiopulmonary, physical, capnometric, and blood gas related parameters were recorded. Breathing resistance and work of breathing were significantly increased while wearing a mask. During exercise the increase in minute ventilation tended to be lower and breathing time was significantly longer with mask than without mask. Wearing a mask caused significant minimal decreases in blood oxygen pressure, oxygen saturation, an initial increase in blood and inspiratory carbon dioxide pressure, and a higher perceived physical exertion and temperature and humidity behind the mask under very heavy exercise. All effects were stronger when wearing an FFP2. Wearing face masks at rest and under exercise, changed breathing patterns in the sense of physiological compensation without representing a health risk. Wearing a mask for 4-h during light work had no effect on blood gases.

## Introduction

Face masks have been confirmed as adequate protection against SARS-CoV-2 infection^1–3^. In many countries, wearing a mask is required in public and at workplaces, especially if social distancing cannot be assured. This study was performed to gain additional knowledge about strains and stresses caused by wearing masks at rest and under different working (exercising) conditions.

Numerous studies showed that wearing masks during physical exercise causes non-health relevant decreases in blood oxygen as well as slight increases in blood carbon dioxide^2,4–10^. However, the majority of these studies focused on relevant clinical parameters, sporting leisure activities and investigated short-term loads of up to 300 watts and more on a bicycle ergometer which does not represent typical conditions in everyday life or at workplaces^11–15^. Some authors also reported physiological effects after continuous or moderate loads^10,15–19^. In most studies, during cardiopulmonary exercise test (CPET), the face mask was worn underneath the silicone mask required for CPET, which has been discussed as influencing factor^4–7,13^. Additionally, measurements were predominantly performed on young, well-trained people^10,14,20–22^ and none of the preceding studies was blinded with respect to the type of mask to be worn by the subjects or focused on breathing physiology or breathing mechanics.

To address the gap of knowledge regarding these questions, we created a partially (two of four modules) double-blinded randomized cross-over design for the present study. The aim of the study was to investigate the influence of three common types of masks worn for protection against SARS-CoV-2 on basic breathing physiology, as well as cardiopulmonary parameters and subjects' perceived physical exertion under different exercise and common workplace conditions in a study group covering a wide range of age and training condition.

We hypothesize that (1) face masks alter the breathing patterns (e.g. longer inspiratory and expiratory time or reduced minute ventilation) when compared to the no mask situation under different exercise as well as common workplace conditions. Further we hypothesize, that (2) wearing face masks has a negative effect on objective (cardiopulmonary, blood gas parameters) and subjective (subjects' perceived physical exertion) outcomes under different exercise and common working conditions.

## Materials and methods

### Subjects

Eligibility criteria included being between 18 and 65 years of age with no (medical or psychosocial) contraindication against vigorous exercise. Exclusion criteria were absolute and relative contraindications for CPET as well as intake of psychoactive substances, renal, neurological, or mental diseases, musculoskeletal disorders or any acute injury, according to current recommendations^23,24^.

Subjects underwent a baseline examination consisting of medical history, physical examination, routine laboratory tests, electrocardiogram, a pulmonary function test, and an initial CPET to determine individual load levels (see module 2).

Sample size calculation (G*Power Ver. 3.9.1, level of alpha = 0.05, power = 0.80) based on different cardiopulmonary parameters (e.g., minute ventilation (VE), heart rate (HR), blood oxygen saturation (sO_2_), transcutaneous CO_2_ (tCO_2_)) were performed using the data of previous studies^8,10,12,15^. The largest number of subjects to be assumed was 39 (exercise with a surgical mask (SM) and without wearing a mask).

### Study design

**Figure 1:**
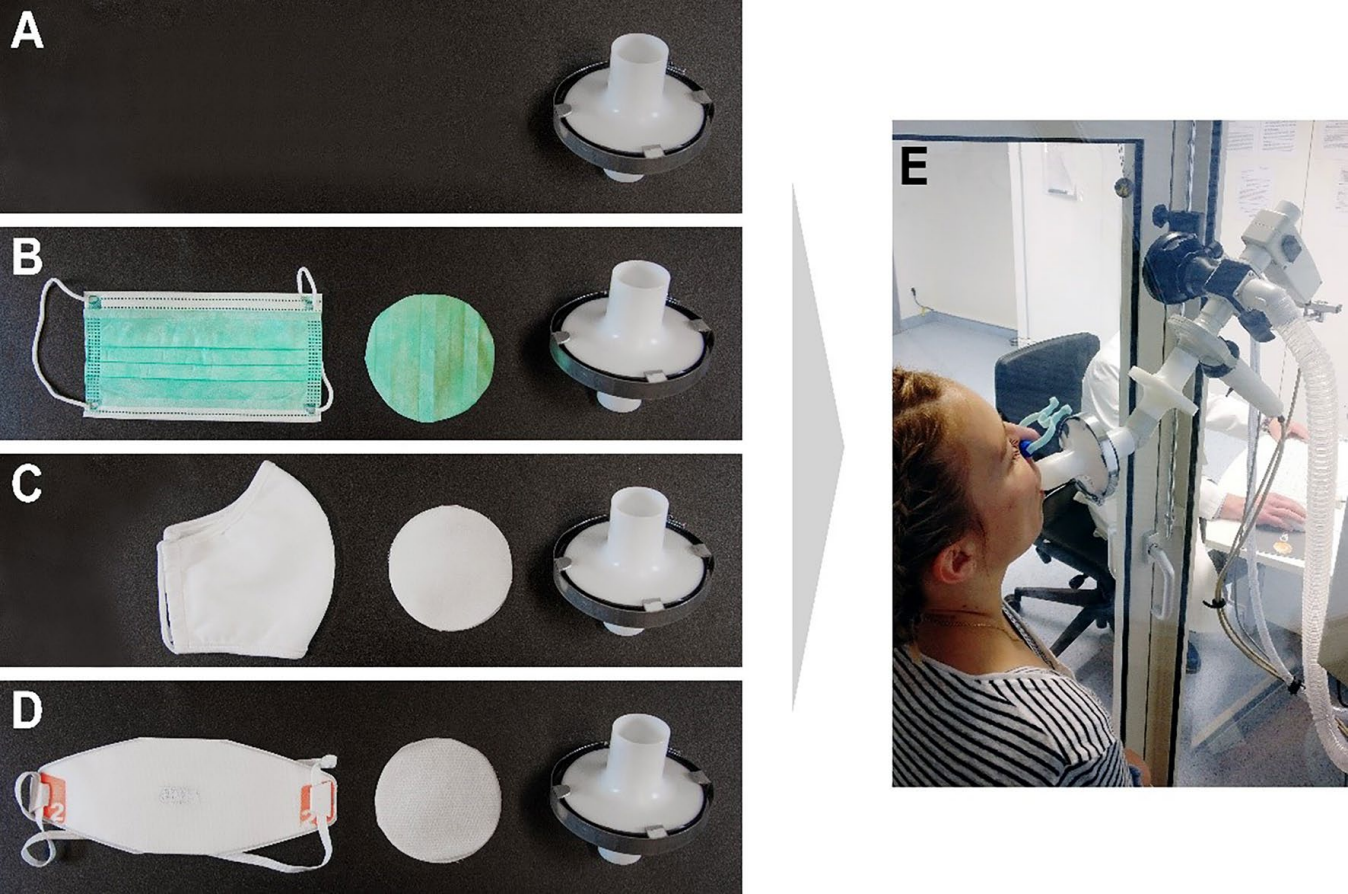
Mask adapter. (**A**) round material sample (Ø 8 cm) of the tested mask (**B** surgical mask, **C** community mask, **D** FFP2) was placed in an opened, empty filter, which was then airtight closed and used for the body plethysmographic (**E**) and spiroergometric (CPET) examinations. For no mask situation (**A**), an empty filter was used.

Each subject was tested with four different mask situations (surgical mask (SM), community cloth mask (CM), a filtering face piece (FFP2) and without wearing a mask (NM) as reference) in a randomized order. The cross-over study design was based on four different modules performed also in a randomized order to investigate different physiological parameters that may be influenced when wearing a face mask: body plethysmography (1) and CPET (2) were performed to study breathing physiology and cardiopulmonary parameters at rest and during exercise under standardized conditions without leakage of the mask. In these two modules the mask material was presented to subjects in a double-blinded setting using a mask adapter (Fig. 1) to avoid influences of wearing the face mask behind the silicone CPET mask and to reduce bias of the subjects' perceived physical exertion and the experimental conditions observed by the investigator. The modules ergometry (3) and workplace examination (4) were performed to study the influence of masks worn under real-life conditions (including leakage) especially on blood gases and inspiratory and expiratory CO_2_ partial pressure under exercise and common work conditions.

In module 1, the washout periods between each mask situations lasted at least 30 min. If the subjects were not ready for a subsequent intervention, they were given an additional 30 min resting time. In modules 2–3, the washout periods between each exercise test lasted at least 5 h. Blood gas samples was drawn to control if subjects had regained a physiological resting state. For module 4, the washout period between each mask situations was more than 48 h. In all four modules, each session was performed at a comparable time of the day with comparable routines (i.e. working days).

### Modules

#### Module 1

Body plethysmography measurements (MasterScreen Body®, Vyaire Medical GmbH, Höchberg, Germany) were performed as described previously^17,18^ and all data were generated via the company software (JLAB 5.30.0, Vyaire Medical GmbH, Höchberg, Germany) and according to current recommendations^16–18^.

#### Module 2

CPET (MasterScreen CPX®, Vyaire Medical GmbH, Höchberg, Germany) was performed using a standard silicone CPET mask (Hans Rudolph™ mask) on a bicycle ergometer according to current recommendations^23,24^. Based on an initial CPET, individually determined load levels resulting in a VE of 10 L/min (pre), 30 L/min (E1), 50 L/min (E2), > 60 L/min (E3) and 10 L/min (post) were used, which corresponds to light (pre and post), moderate (E1), heavy (E2), and very heavy (E3) work^25,26^. To assure a physiological steady-state situation, we decided to use load levels with 6 min duration, which represent a more sensitive and reliable verification of constant load thresholds (e.g. physical working capacity at a heart rate of 130/150 beats per minute (PWC_130/150_)) than extrapolation from a ramp load (< 3 min at each load level)^27,28^. Each exercise test took about 30 min.

Before each examination (modules 1 and 2), the measuring device was calibrated with the specific mask adapter and the specific dead-space volume (consisting of the volumes of the mask adapter and of the CPET mask) was recorded in the system. However, this dead-space volume was very similar to the dead-space volume of any face mask worn under real-life conditions (modules 3 and 4).

#### Module 3

Since the CPET examination does not reflect the normal wearing of a face mask (without leakage), due to the standard silicone CPET mask, ergometric exercise was repeated using the identical step protocol as in module 2 and the masks were worn under real-life conditions (including leakage).

#### Module 4

For a 4-h workplace examination, masks were regularly worn (including leakage) during light/moderate work in the office or laboratory. For modules 3 and 4, the correct fit of the mask was checked by the investigator prior to each measurement and the subjects were instructed to do the same during the workplace measurement in module 4.

### Mask characteristics and mask adapter

To compare breathing resistances and filter efficiencies, the three masks were tested on a “Sheffield head” according to the European Standard EN 149 (applied solely to CE certification of FFP masks)^29^.

In all modules, participants wore in randomized order (1) NM, (2) SM (Typ II, MedicalCare & Serve industry®, Wilfried Rosbach GmbH, Willich, Germany), (3) CM (van Laack® GmbH, Mönchengladbach, Germany) and (4) FFP2 (Dräger X-plore® 1920 NR D, Dräger® Safety AG, Lübeck, Germany). For body plethysmography (Fig. 1E) and CPET, a round sample (**⌀** 8 cm) of the tested mask was placed in an opened, empty bacterial filter (MicroGard II, Vyaire Medical GmbH, Höchberg, Germany), made airtight using adhesive tape and a metal clamp (Fig. 1 B–D). The original filter as well as the filter housing are explicitly approved and required by the manufacturer for body plethysmography and CPET measurements (Vyaire Medical GmbH, Höchberg, Germany). For the NM situation, an empty filter was prepared the same way, assuring a double-blind experimental setting (Fig. 1A).

### Physiological and physical parameters

Lung volume associated parameters (e.g. forced vital capacity (FVC), forced expiratory volume in one second (FEV_1_)), as well as parameters referring to breathing time (inspiratory time (Tin), expiratory time (Tex), inspiratory and expiratory time (Ttot)) and parameters related to body plethysmography (e.g., total airway resistance (Rtot), specific airway resistance (sRtot), work of breathing (WOB), respiratory power (RP)) were measured via body plethysmography under resting situation according to current recommendations^23,30,31^.

Blood gas analysis (BGA) from hyperaemic capillary earlobe blood was taken before and after CPET and ergometry and at the end of each load level (5th minute) as well as before and at the end (while wearing a mask) of the 4-h workplace examination (ABL 825 analyser, Radiometer Medical APS, Copenhagen, Denmark) to check sO_2_, oxygen partial pressure (pO_2_), and blood carbon dioxide partial pressure (pCO_2_).

During CPET, cardiopulmonary parameters such as minute ventilation (VE), respiratory rate (RR), heart rate (HR), oxygen uptake (VO_2_), minute ventilation per litre of oxygen (VE/VO_2_) and carbon dioxide (VE/VCO_2_), and parameters referring to breathing time (Tin, Tex, Ttot) were measured automatically via CPET breath by breath, while blood pressure was monitored every two minutes with a cuff sphygmomanometer (Ergoselect 200, Ergoline GmbH, Bitz, Germany) according to current recommendations^23,24^.

For ergometry and workplace examination, the heart rate (HR) and respiratory rate (RR) were measured simultaneously using a portable, wireless polysomnography system (SOMNOscreen® Plus PSG, SOMNOmedics GmbH, Randersacker, Germany).

According to current recommendations, changes in inspiratory and expiratory CO_2_ partial pressure (pCO_2_in, pCO_2_ex) were performed using the LoFLo capnography system (Philips Respironics, Wallingford Connecticut, USA) with a sampling rate of 50 ml per minute^32^.

Temperature and humidity were recorded using a climate data logger (PeakTech 5185®, Ahrensburg, Germany), which was fixed with adhesive tape between nose and mouth^33,34^.

### Subjects’ perceived physical exertion

Perceived physical exertion was assessed in CPET and ergometry before, after, and within the last 20 s of each load as well as before and after the 4-h workplace examination using the BORG Scale^35^.

### Data and statistical analysis

Median values of cardiopulmonary parameters of CPET and ergometry were calculated during the last three minutes of each 6-min load after reaching the physiological steady state. Measurement values are presented as boxplots (box: median, 25th-75th percentile; whiskers: 3.5-fold interquartile range). In tables, data are expressed as medians (range).

A generalized linear mixed (GLM) model, along with generalized estimating equations (GEE), was used on the logarithmized values. In all GLM models, the load level (pre, E1, E2, E3, post) and the time of measurement (30, 60, 90, 120, 150, 180, 210, 240 min) were included as influencing factors. This allows intraindividual comparison at the different examination times (i.e., each subject is compared to him/herself), and a consideration of repeated measurements. Geometric means (G.M.) as well as least-square means were calculated based on these models. The situation without mask was used as reference. We adjusted for further influencing factors, including sex (dichotomous), age (continuous per 10 years), and height (continuous per 10 cm). Pearson correlation coefficients (r) (+ 95% confidence intervals (CI) and Fisher transformation over all examination times in each module) were calculated to predict the monotone association between parameters. A *p* value of < 0.05 was considered statistically significant. Analyses were performed using SAS 9.4 (SAS Institute, Cary, NC, USA). Figures were drafted with GraphPad Prism version 9 (GraphPad Software, San Diego, CA, USA).

### Ethic approval

The study was conducted in accordance with the latest revision of the Declaration of Helsinki and the Ethics Committee of the medical faculty of the Ruhr-University Bochum provided ethical approval of the study (Reg. No.: 20-7024). All subjects gave written informed consent—both for study participation and for the publication. The person shown in Fig. 1 gave informed consent for publication of identifying images in an open access-online publication.

## Results

### Performance characteristics of the face masks

Using the “Sheffield head” system according to EN 149, in- and expiratory breathing resistance of SM and CM was similar and lower compared to FFP2, probably due to the better filter efficiency of FFP2 (Table S1). For all types of masks breathing resistance increased with higher inspiratory airflow and decreased for SM and CM under an expiratory airflow from 160 L/min due to a high leakage of the masks. The breathing resistance of FFP2 increased further, since the leakage was low. The filter efficiency was highest for FFP2 and much lower for CM and SM as expected.

### Characteristics of the study cohort

A total of 40 subjects (20 women and 20 men) participated in the study. Data are shown in Table 1. Mean age for the study group was 47 years (range 19–65). Age, weight, height, BMI were lower in females compared to males. Training conditions (PWC_130_) were in the normal range. The cohort included 8 active smokers and some subjects suffering from mild asthma (n = 2) or hypertension (n = 5). All 40 subjects completed the four modules without showing clinical adverse health effects.

**Table 1:** Characteristics of the study participants (median and range).

	All N = 40	Men N = 20	Women N = 20
Age (years: median (range))	47 (19–65)	49 (19–65)	44 (23–61)
Height (cm: median (range))	180 (160–196)	185 (175–196)	170 (160–182)
Weight (kg: median (range))	75 (57–121)	85 (72–121)	68 (57–90)
BMI (kg/m^2^: median (range))	24.2 (19.9–34.6)	24.5 (21.3–34.6)	23.8 (19.9–31.3)
PWC_130_ (W/kg: median (range))	1.52 (0.88–2.18)	1.55 (1.01–2.11)	1.53 (0.88–2.18)
Smokers (n)	8	2	6
Former smokers (n)	13	7	6
Mild asthma (n)	2	2	0
Hypertension (n)	5	3	2

*BMI* Body-mass index, *PWC*_*130*_ Physical working capacity at a heart rate of 130 beats per minute.

### Body plethysmography (Module 1)

When wearing a mask, forced vital capacity (FVC) and forced expiratory volume in one second (FEV1) decreased significantly (Table 2). Compared with the situation without mask, body plethysmography showed a significant increase in breathing resistance, work of breathing, and respiratory power depending on the mask type (SM < CM < FFP2), resulting in a significant reduction of the maximum voluntary ventilation (MVV). Inspiratory and expiratory time (Ttot) increased, indicating a reduced breathing frequency and a slower and deeper single breath.

**Table 2:** Body plethysmography results of 40 subjects tested without mask (NM) and with three different mask types (SM, CM, FFP2) using the mask adapter.

	Measured results				Results of GLM model			
	NM median range	SM median range	CM median range	FFP2 median range	NM geometric mean	SM Δ *p* value	CM Δ *p* value	FFP2 Δ *p* value
VC (L)	4.67 2.74–7.30	4.67 2.79–7.39	4.74 2.69–7.34	4.66 2.80–7.33	4.62	− 0.1 0.165	− 0.1 0.311	− 0.1 0.274
FVC (L)	4.82 2.76–7.27	4.69 2.65–7.23	4.77 2.67–7.33	4.73 2.83–7.19	4.67	− 0.08 ** < 0.001**	− 0.09 ** < 0.001**	− 0.09 ** < 0.001**
FEV_1_ (L)	3.84 2.09–5.65	3.76 2.08–5.75	3.72 2.01–5.66	3.69 2.13–5.67	3.73	− 0.07 **0.003**	− 0.10 ** < 0.001**	− 0.09 ** < 0.001**
FEV_1_/FVC (%)	79.79 65.13–93.24	78.45 65.00–93.24	77.81 62.01–92.82	77.33 63.79–92.72	79.39	− 0.39 0.227	− 0.92 **0.005**	− 0.90 **0.006**
PEF (L*s^−1^)	8.77 4.59–12.75	8.32 4.34–13.21	7.93 4.46–11.65	7.94 4.08–11.52	8.36	− 0.27 **0.037**	− 0.54 ** < 0.001**	− 0.63 ** < 0.001**
MVV (L*min^−1^)	115 63–170	113 62–173	111 60–170	111 64–170	111.78	− 1.93 **0.003**	− 2.82 ** < 0.001**	− 2.61 ** < 0.001**
Rtot (kPa*s*L^−1^)	0.15 0.07–0.28	0.19 0.04–0.34	0.21 0.03–0.33	0.28 0.07–0.44	0.14	0.05 ** < 0.001**	0.06 ** < 0.001**	0.11 ** < 0.001**
sRtot (kPa*s)	0.5 0.3–1.4	0.7 0.3–1.7	0.8 0.4–1.5	1.0 0.2–2.2	0.64	− 0.20 ** < 0.001**	0.28 ** < 0.001**	0.50 ** < 0.001**
WOB (kPa*L)	0.21 0.04–0.63	0.25 0.05–0.88	0.26 0.11–1.18	0.27 0.05–0.96	0.20	0.05 **0.001**	0.08 ** < 0.001**	0.09 ** < 0.001**
RP (Watt)	0.07 0.01–0.20	0.07 0.02–0.22	0.09 0.03–0.41	0.08 0.02–0.33	0.06	0.02 **0.005**	0.03 ** < 0.001**	0.03 ** < 0.001**
Ttot (s)	3.27 1.78–4.83	3.28 2.28–5.14	3.40 2.24–5.69	3.47 2.33–6.08	3.24	0.08 0.324	0.03 0.708	0.09 0.297

*CM* Community mask, *Δ* Difference to NM, *FEV*_*1*_ Forced expiratory volume in 1 s, *FEV1% VCMAX (%)* Tiffeneau Index, *FFP2* Filtering face piece class 2, *FVC* Forced vital capacity, *MVV* Maximum voluntary ventilation (FEV_1_ *35), *NM* No mask, *RP* Respiratory power, *Rtot* Total airway resistance, *SM* Surgical mask, *sRtot* Specific airway resistance, *PEF* Peak expiratory flow, *Ttot* Inspiratory and expiratory time, *VC* Ventilation capacity, *WOB* Work of breathing, *WOBin* Inspiratory work of breathing, *WOBex* Expiratory work of breathing.

Presented are measured results (median, range). Differences (Δ) of NM to SM, CM, and FFP2 were analysed using a generalized linear mixed model (GLM).

Significant values are in bold.

### CPET (Module 2)

Main results of CPET are displayed in Table 3 (full results in Supplementary Tables S2 and S3). Blood-gas analyses revealed a slight decrease in pO_2_ and sO_2_ as well as a slight increase in pCO_2_ under light work depending on the mask type (SM < CM < FFP2). All effects were most pronounced when wearing FFP2.

**Table 3:** CPET results of generalized linear mixed model (GLM model) analysis of 40 subjects without mask (NM) and with three different mask types (SM, CM, FFP2).

	Pre (light work)				E1 (moderate work)				E2 (heavy work)				E3 (very heavy work)				Post (light work)			
	NM	SM	CM	FFP2	NM	SM	CM	FFP2	NM	SM	CM	FFP2	NM	SM	CM	FFP2	NM	SM	CM	FFP2
	G.M	Δ *p* value			G.M	Δ *p* value			G.M	Δ *p* value			G.M	Δ *p* value			G.M	Δ *p* value		
Subjects’ perceived physical exertion																				
BORG (0–10)	0.81	− 0.05	− 0.04	− 0.18	0.96	0.05 0.651	0.22 0.306	0.33 **0.022**	2.14	0.33 0.384	0.23 0.522	0.89 **0.008**	3.68	0.59 0.360	0.54 0.419	1.21 **0.016**	0.88	0.11 0.498	0.14 0.475	0.22 0.064
Pulmonary parameters																				
Ttot (s)	3.91	− 0.08	− 0.04	0.18	2.95	0.08 0.491	− 0.01 0.707	0.23 0.130	2.53	0.04 0.251	0.00 0.463	0.22 0.333	2.11	0.06 0.171	0.08 0.197	0.23 0.124	2.95	0.03 0.399	− 0.03 0.944	0.11 0.848
RR (min^−1^)	15.37	0.01	0.17	− 0.85	20.31	− 0.51 0.521	0.14 0.918	− 1.52 0.610	23.71	− 0.38 0.670	0.01 0.794	− 2.04 0.476	28.37	− 0.76 0.450	− 0.97 0.220	− 2.70 0.243	20.43	− 0.38 0.622	0.09 0.869	− 0.85 0.731
VT (L)	0.90	0	− 0.01	− 0.02	1.64	− 0.01 0.948	− 0.04 0.662	− 0.01 0.518	1.98	0 0.922	− 0.03 0.792	0 0.553	2.25	− 0.01 0.938	0 0.720	− 0.03 0.353	1.25	− 0.02 0.709	− 0.05 0.440	0 0.599
VE (L*min^−1^)	12.03	0.28	0.26	− 0.42	31.26	− 0.40 0.380	− 0.39 0.407	− 1.91 0.506	45.47	− 0.67 0.344	− 0.93 0.293	− 3.95 0.164	62.52	− 1.93 0.187	− 2.51 0.129	− 7.19 **0.035**	23.3	− 0.88 0.225	− 0.65 0.328	− 0.92 0.937
VO_2_ (ml*min^−1^)	301.78	− 27.34	− 21.06	− 36.51	1050.92	− 19.36 0.095	− 36.41 0.367	− 73.39 0.251	1474.55	− 21.65 0.067	− 33.02 0.217	− 92.81 0.168	1910.85	− 38.02 0.076	− 39.58 0.191	− 124.26 0.176	475.16	35.8 0.858	43.45 0.838	43.78 0.621
VCO_2_ (ml*min^−1^)	243.71	− 20.26	− 14.02	− 23.12	889.26	− 29.53 0.264	− 32.85 0.649	− 59.74 0.525	1361.39	− 36.30 0.198	− 32.13 0.446	− 94.92 0.555	1850.06	− 42.07 0.184	− 38.13 0.424	− 122.94 0.520	526.86	− 48.50 0.881	− 40.13 0.759	− 36.44 0.668
VE/VO_2_	39.87	4.96	3.89	3.87	29.74	0.17 ** < 0.001**	0.68 **0.014**	0.28 **0.004**	30.83	0 ** < 0.001**	0.07 **0.001**	− 0.79 ** < 0.001**	32.71	− 0.37 ** < 0.001**	− 0.66 ** < 0.001**	− 1.76 ** < 0.001**	49.03	− 5.17 0.086	− 5.37 0.116	− 5.92 0.096
VE/VCO_2_	49.36	5.70	4.11	3.24	35.15	0.74 ** < 0.001**	0.88 **0.035**	0.23 **0.030**	33.39	0.41 ** < 0.001**	0.11 **0.003**	− 0.62 **0.002**	33.78	− 0.27 ** < 0.001**	− 0.67 ** < 0.001**	− 1.69 ** < 0.001**	44.20	2.66 0.094	2.33 0.343	1.43 0.295
EELV (L)	3.98	0.20	0.37	0.02	3.88	0.06 0.413	− 0.01 0.129	0.32 0.920	3.81	0.09 0.758	0.15 0.540	− 0.10 0.698	3.94	− 0.30 0.125	− 0.29 0.064	− 0.43 0.142	4.28	− 0.06 0.458	− 0.38 **0.034**	− 0.71 **0.031**
BR (%)	10.85	0.44	0.51	− 0.13	28.15	0.16 0.400	0.40 0.427	− 1.05 0.528	40.93	0.16 0.365	0.27 0.313	− 2.59 0.178	56.28	− 0.70 0.201	− 0.78 0.140	− 5.19 **0.039**	20.98	− 0.41 0.236	− 0.03 0.343	− 0.31 0.957
Metabolic parameters																				
pH	7.41	0.00	0.00	0.00	7.40	− 0.01 0.756	− 0.01 0.932	− 0.01 0.930	7.39	− 0.01 0.623	− 0.01 0.967	− 0.01 0.380	7.37	− 0.02 0.252	− 0.01 0.904	− 0.02 **0.027**	7.38	− 0.01 0.381	− 0.01 0.581	− 0.01 0.428
pCO_2_ (mmHg)	36.13	0.65	0.67	1.33	37.77	0.83 0.845	0.53 0.802	1.40 0.995	37.80	0.46 0.748	0.31 0.573	1.72 0.663	35.94	1.23 0.397	1.05 0.590	2.39 0.133	33.99	0.50 0.866	0.65 0.985	1.55 0.664
pO_2_ (mmHg)	90.79	− 3.58	− 1.00	− 2.18	92.17	− 2.82 0.640	− 1.15 0.942	− 2.21 0.994	91.16	− 2.82 0.472	− 1.15 0.712	− 2.21 0.891	89.45	− 2.92 0.701	− 2.70 0.290	− 2.22 0.965	94.03	− 1.00 0.137	− 0.38 0.727	− 0.05 0.230
Lactate (mmol/L)	1.22	0.00	0.01	0.04	1.61	− 0.02 0.774	0.01 0.895	0.10 0.780	2.51	0.04 0.896	0.00 0.860	0.12 0.883	4.58	0.03 0.987	− 0.11 0.616	0.09 0.826	3.79	0.04 0.929	− 0.06 0.698	0.05 0.761
sO_2_ (%)	97.17	− 0.34	− 0.18	− 0.33	97.27	− 0.32 0.947	− 0.18 0.959	− 0.27 0.730	96.87	− 0.25 0.643	− 0.08 0.603	− 0.21 0.531	96.72	− 0.46 0.528	− 0.35 0.382	− 0.53 0.309	97.17	− 0.12 0.282	− 0.03 0.474	− 0.08 0.209

*BORG* Borg scale, *BR* Breathing reserve, *CM* Community mask, *CPET* Cardiopulmonary exercise test (spiroergometry), *Δ* Difference to NM, *EELV* End-expiratory lung volume, *FFP2* Filtering face piece class 2, *G.M.* Geometric mean, *NM* No mask, *pCO*_*2*_ Partial pressure of carbon dioxide, *pO*_*2*_ Partial pressure of oxygen, *pH* Potential of hydrogen in blood, *RR* Respiratory rate, *sO*_*2*_ Haemoglobin oxygen saturation, *SM* Surgical mask, *Tin* Inspiratory time, *Tex* Expiratory time, *Ttot* Inspiratory and expiratory time, *VCO*_*2*_ Carbon dioxide production, *VE* Minute ventilation, *VE/VCO*_*2*_ Ventilation per litre of carbon dioxide, *VE/VO*_*2*_ Ventilation per litre of oxygen, *VO*_*2*_ Oxygen uptake, *VT* Tidal volume.

Masks were tested using the mask adapter (details see Methods). The situation without mask in each specific load level (pre, E1, E2, E3, post) was always used as reference. For NM geometric mean (G.M.), for the three mask types (SM, CM, FFP2) differences (Δ) to NM and *p* values are shown.

Significant values are in bold.

**Figure 2:**
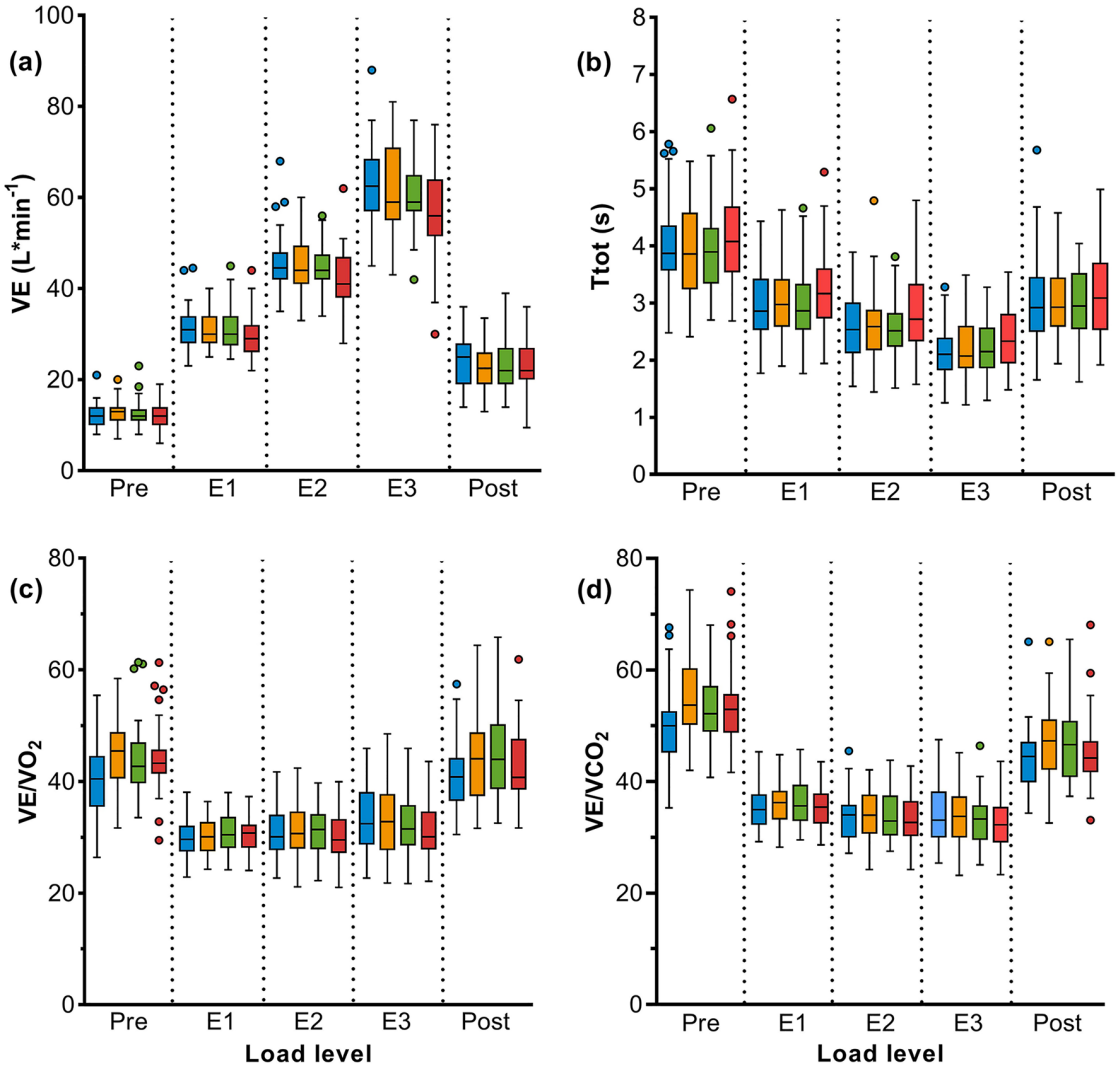
Cardiopulmonary exercise test measurement: Results of (**a**) minute ventilation (VE), (**b**) inspiratory and expiratory time (Ttot), (**c**) ventilation per litre of oxygen (VE/VO_2_), and (**d**) ventilation per litre of carbon dioxide (VE/VCO_2_) in 40 subjects without mask (blue), with surgical mask (yellow), community mask (green), and FFP2 (red). The load levels correspond to light work (pre and post), moderate (E1), heavy (E2) and very heavy work (E3). P-values are shown in Table 3.

The increase in minute ventilation (VE) tended to be lower when wearing a mask than without mask, reaching statistical significance with FFP2 during very heavy work (E3) (Fig. 2a, Table 3). Under very heavy work, end-expiratory lung volume (EELV) and breathing reserve (BR) were lower with all types of mask compared to NM. For breathing reserve, the difference was statistically significant, when wearing FFP2. With each type of mask, oxygen uptake (VO_2_) was reduced and Ttot was longer with increasing load levels than without wearing a mask, especially for FFP2 under very heavy work (Fig. 2b, Table 3).

VE/VO_2_ and VE/VCO_2_ increased during light work (E1) while wearing a mask, but decreased significantly under moderate exercise (E2) for FFP2. At very heavy load (E3) VE/VO_2_ and VE/VCO_2_ were reduced significantly for all types of masks (Fig. 2c, d, Table 3).

Further analyses showed minor positive correlations for all mask types between FVC, inspiratory and expiratory time (Ttot), and FEV_1_, respectively (Supplementary Table S4). Both parameters (FVC, FEV_1_) showed negative correlations with VE/VO_2_ and VE/VCO_2_ under exercise. In addition, for SM, CM and FFP2 there was a negative correlation between FVC or FEV_1_ and BORG scale and a positive correlation of specific airway resistance with Ttot. With longer Ttot, pCO_2_ increased and pO_2_ decreased for all types of masks. However, for FFP2, a minor negative correlation was observed between Ttot and sO_2_.

### Ergometry (Module 3)

**Figure 3:**
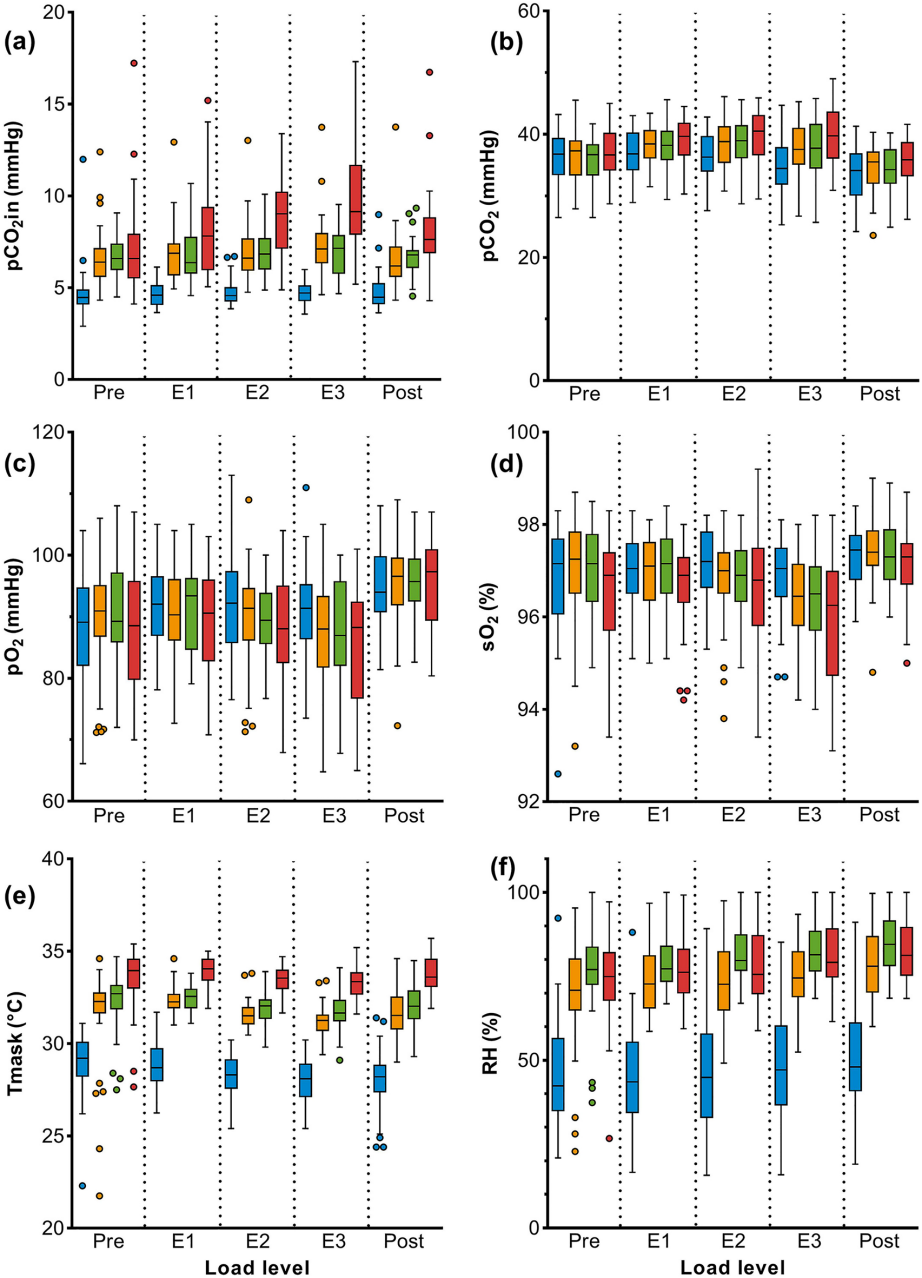
Ergometry measurements: Results of (a) inspiratory CO_2_ behind the mask (pCO_2_in), (**b**) blood partial pressure of CO_2_ (pCO_2_); (**c**) blood partial pressure of O_2_ (pO_2_); (**d**) haemoglobin oxygen saturation (sO_2_), (**e**) temperature behind the mask (Tmask), and (**f**) relative humidity behind the mask (RH) in 40 subjects wearing no mask (blue), surgical mask (yellow), community mask (green), and FFP2 (red). The load levels correspond to light work (pre and post), moderate (E1), heavy (E2) and very heavy work (E3). P-values are shown in Table 4.

Main results of ergometry are displayed in Table 4 (complete dataset in Table S5). With mask, Ttot was longer with increasing load than without mask, which was most evident with the FFP2. Inspiratory CO_2_ behind the mask (pCO_2_in) increased at rest and under physical exertion, being significant for FFP2 (Fig. 3a). Blood-gas analyses showed a significant increase in pCO_2_ with higher exercise levels for all types of masks compared to NM (Fig. 3b). In the post-exercise phase, CO_2_ concentration decreased immediately and was even slightly lower compared to the pre-exercise phase. Significant decreases in pH, pO_2_ and sO_2_ were measured at very high workloads (E3) with all masks but remained predominantly within physiological reference limits (Fig. 3c, d). In some subjects (n = 7), there was a decrease under heavy exercise to the lower physiological limit (Fig. 3c, d). In the post-exercise phase, pO_2_ and sO_2_ returned quickly to normal and tended to be higher than in the pre-exercise phase.

**Table 4:** Ergometry results of generalized linear mixed model (GLM model) analysis of 40 subjects without mask (NM) and with three different mask types (SM, CM, FFP2).

	Pre (light work)				E1 (moderate work)				E2 (heavy work)				E3 (very heavy work)				Post (light work)			
	NM	SM	CM	FFP2	NM	SM	CM	FFP2	NM	SM	CM	FFP2	NM	SM	CM	FFP2	NM	SM	CM	FFP2
	G.M	Δ *p* value			G.M	Δ *p* value			G.M	Δ *p* value			G.M	Δ *p* value			G.M	Δ *p* value		
Subjects’ perceived physical exertion																				
BORG (0–10)	0.81	− 0.05	− 0.04	− 0.18	0.96	0.05 0.651	0.22 0.306	0.33 0.022	2.14	0.33 0.384	0.23 0.522	0.89 **0.008**	3.68	0.59 0.360	0.54 0.419	1.21 **0.016**	0.88	0.11 0.498	0.14 0.475	0.22 0.064
Pulmonary parameters																				
Ttot (s)	2.90	0.31	0.31	0.6	2.45	0.14 0.239	0.20 0.577	0.20 **0.007**	2.28	0.03 **0.012**	0.05 **0.022**	0.04 ** < 0.001**	1.99	0.03 **0.018**	0.06 **0.040**	0.04 ** < 0.001**	2.44	0.15 0.250	0.13 0.176	0.07 ** < 0.001**
RR (min^1^)	19.48	− 1.75	− 1.51	− 3.06	23.69	− 1.20 0.257	− 1.62 0.791	− 1.89 **0.018**	25.69	− 0.18 **0.010**	− 0.30 **0.040**	− 0.5 ** < 0.001**	29.72	− 0.46 **0.023**	− 0.74 **0.107**	− 0.74 ** < 0.001**	23.69	− 1.64 0.543	− 1.01 0.312	− 0.67 ** < 0.001**
Metabolic parameters																				
pH	7.42	0.00	− 0.01	− 0.01	7.41	− 0.01 0.109	− 0.02 0.096	− 0.02 0.158	7.40	− 0.02 **0.060**	− 0.02 **0.030**	− 0.02 0.163	7.39	− 0.03 **0.001**	− 0.03 **0.001**	− 0.04 ** < 0.001**	7.40	− 0.02 0.063	− 0.03 **0.010**	− 0.03 0.056
pCO_2_ (mmHg)	36.01	0.31	− 0.22	0.50	36.69	1.60 0.079	1.31 **0.033**	2.09 **0.033**	36.36	1.93 **0.023**	2.17 **0.001**	2.92 **0.001**	34.58	3.04 ** < 0.001**	2.71 ** < 0.001**	4.71 ** < 0.001**	33.28	1.05 0.230	0.97 0.062	2.00 **0.017**
pO_2_ (mmHg)	87.86	1.81	1.33	− 0.41	91.53	− 1.40 0.141	− 0.64 0.365	− 2.90 0.259	91.77	− 1.86 0.081	− 2.36 0.079	− 3.62 0.130	90.27	− 3.45 **0.008**	− 2.70 **0.041**	− 5.66 **0.007**	95.00	0.17 0.438	0.69 0.744	0.20 0.774
Lactate (mmol/L)	1.36	0.00	− 0.08	− 0.03	1.69	− 0.07 0.621	− 0.05 0.715	− 0.09 0.672	2.51	− 0.03 0.864	0.01 0.435	− 0.10 0.798	4.50	− 0.13 0.688	− 0.06 0.561	− 0.07 0.971	3.61	0.00 0.980	0.09 0.294	0.02 0.767
sO_2_ (%)	97.17	0.12	0.17	− 0.17	97.27	− 0.11 0.365	− 0.02 0.468	− 0.32 0.572	96.87	− 0.39 **0.049**	− 0.31 0.061	− 0.60 0.098	96.72	− 0.78 **0.001**	− 0.70 **0.001**	− 1.34 ** < 0.001**	97.17	0.13 0.980	0.04 0.594	− 0.16 0.956
pH	7.42	0.00	− 0.01	− 0.01	7.41	− 0.01 0.109	− 0.02 0.096	− 0.02 0.158	7.40	− 0.02 **0.060**	− 0.02 **0.030**	− 0.02 0.163	7.39	− 0.03 **0.001**	− 0.03 **0.001**	− 0.04 ** < 0.001**	7.40	− 0.02 0.063	− 0.03 **0.010**	− 0.03 0.056
pCO_2_ (mmHg)	36.01	0.31	− 0.22	0.50	36.69	1.60 0.079	1.31 **0.033**	2.09 **0.033**	36.36	1.93 **0.023**	2.17 **0.001**	2.92 **0.001**	34.58	3.04 ** < 0.001**	2.71 ** < 0.001**	4.71 ** < 0.001**	33.28	1.05 0.230	0.97 0.062	2.00 **0.017**
pO_2_ (mmHg)	87.86	1.81	1.33	− 0.41	91.53	− 1.40 0.141	− 0.64 0.365	− 2.90 0.259	91.77	− 1.86 0.081	− 2.36 0.079	− 3.62 0.130	90.27	− 3.45 **0.008**	− 2.70 **0.041**	− 5.66 **0.007**	95.00	0.17 0.438	0.69 0.744	0.20 0.774
Hemodynamic parameters																				
HR (min^− 1^)	82.64	0.69	− 0.24	2.56	101.92	1.02 0.942	0.98 0.576	2.65 0.827	121.37	1.14 0.963	− 0.10 0.927	2.23 0.573	140.46	1.48 0.942	− 0.90 0.899	1.56 0.498	101.28	2.26 0.548	0.28 0.806	3.81 0.778
SBP (mmHg)	109.31	1.97	− 0.41	0.70	117.55	4.08 0.589	3.51 0.270	2.37 0.652	138.95	2.67 0.969	2.27 0.485	4.04 0.434	155.93	4.80 0.668	4.01 0.315	6.32 0.253	138.34	0.92 0.744	− 1.76 0.792	4.30 0.477
DBP (mmHg)	76.53	0.95	− 0.10	1.05	72.60	2.57 0.501	0.20 0.903	− 2.46 0.153	75.08	1.26 0.899	− 0.77 0.782	1.56 0.837	75.51	2.23 0.624	1.34 0.579	1.74 0.791	73.43	− 0.07 0.676	− 1.06 0.675	1.14 0.956
Capnometric parameters																				
pCO_2_in (mmHg)	1.63	0.75	0.86	0.99	1.83	0.57 0.680	0.57 0.947	1.06 **0.021**	1.89	0.60 0.643	0.65 0.793	1.46 ** < 0.001**	1.84	0.70 0.281	0.68 0.774	1.92 ** < 0.001**	1.83	0.52 0.243	0.66 0.500	1.33 0.061
pCO_2_ex (mmHg)	26.76	2.14	1.78	2.99	30.00	2.70 0.669	2.28 0.685	3.25 0.901	30.12	2.81 0.586	2.67 0.352	3.31 0.942	28.24	3.81 **0.029**	3.52 **0.019**	4.98 **0.013**	26.58	2.23 0.879	1.85 0.893	2.59 0.597
Mask microclimate																				
Tmask (°C)	28.93	2.54	3.30	4.57	28.81	3.52 **0.049**	3.63 0.499	5.12 0.284	28.22	3.35 0.079	3.68 0.359	5.26 0.131	27.98	3.25 0.111	3.69 0.328	5.36 0.076	27.88	3.72 **0.014**	4.13 **0.071**	5.86 **0.008**
RH (%)	43.56	23.74	31.38	29.28	42.81	29.95 0.101	36.60 0.195	33.24 0.297	41.70	31.12 **0.043**	39.58 **0.040**	35.23 0.105	45.39	29.28 0.289	36.55 0.415	34.08 0.437	48.95	28.42 0.690	35.30 0.992	33.41 0.913

*BORG* Borg scale, *CM* Community mask, *DBP* Diastolic blood pressure, *Δ* Difference to NM, *FFP2* Filtering face piece class 2, *G.M.* Geometric mean, *HR* Heart rate, *NM* No mask, *pCO*_*2*_ Partial pressure of carbon dioxide, pO_2_ Partial pressure of oxygen, *pCO*_*2*_*ex* Expiratory carbon dioxide pressure, *pCO*_*2*_*in* Inspiratory carbon dioxide pressure, *pH* Potential of hydrogen in blood, *RH* Relative humidity, *RR* Respiratory rate, *SBP* Systolic blood pressure, *sO*_*2*_ Haemoglobin oxygen saturation, *SM* Surgical mask, *Tin* Inspiratory time, *Tex* Expiratory time, *Tmask* Temperature behind the mask, *Ttot* Inspiratory and expiratory time.

Masks were worn as in everyday life. The situation without mask in each specific load level (pre, E1, E2, E3, post) was always used as reference. For NM geometric mean (G.M.), for the three mask types (SM, CM, FFP2) differences to NM (Δ) and *p* values are shown.

Significant values are in bold.

Depending on the mask type increased temperature (SM < CM < FFP2) and humidity (SM < FFP2 < CM) were measured behind the mask (Fig. 3e, f).

Further analyses (over all exercise levels in module 3) showed minor positive correlations between FVC and Ttot with a mask (Supplementary Table S6). With longer Ttot, blood pCO_2_ increased and pO_2_ decreased when wearing any mask. However, only for FFP2 a minor negative correlation between Ttot and sO_2_ was found (Supplementary Table S6), which was consistent with the CPET results (Supplementary Table S4).

### Workplace (Module 4)

**Figure 4:**
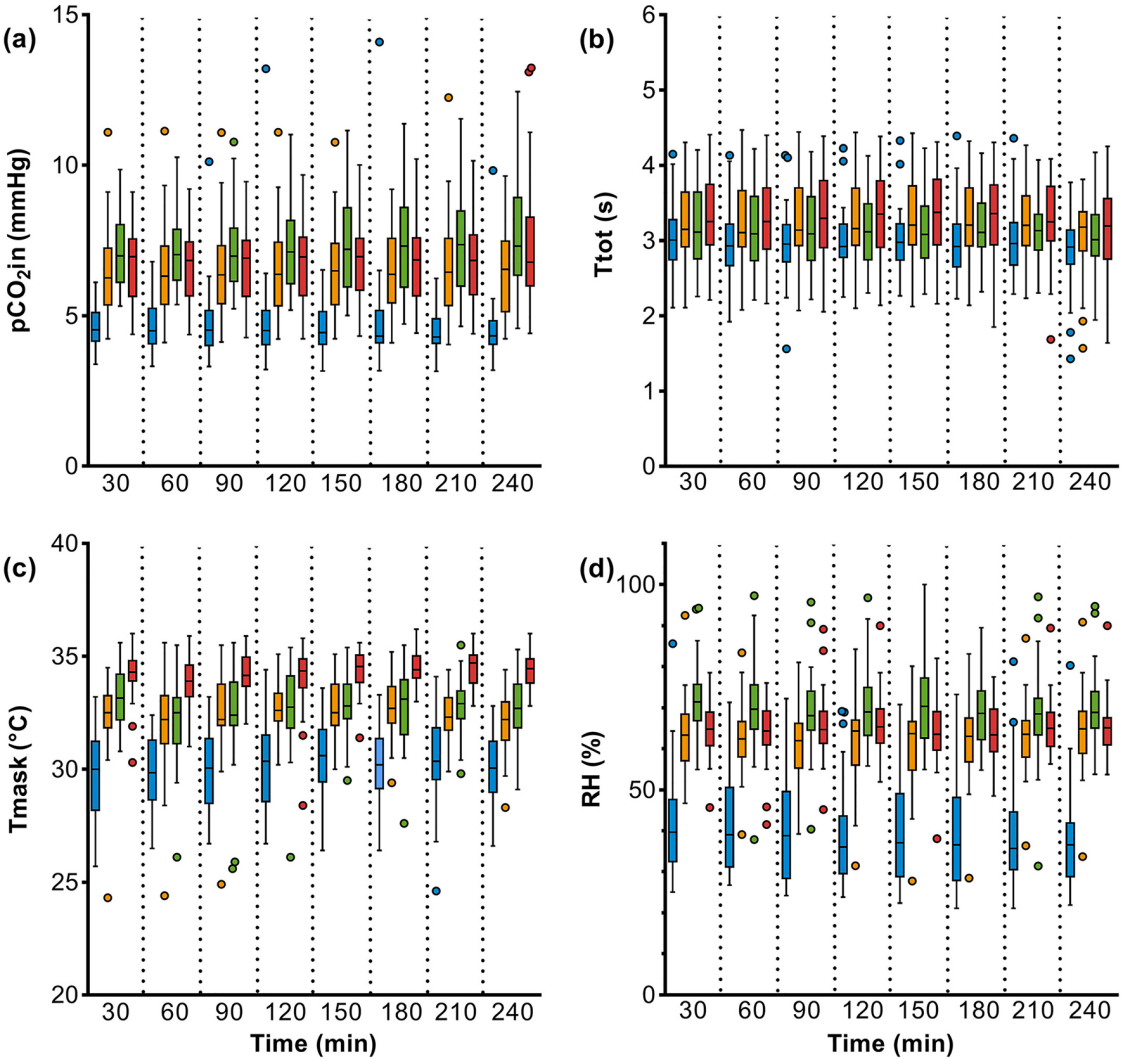
Workplace measurement: Results of (**a**) inspiratory CO_2_ behind the mask (pCO_2_in), (**b**) inspiratory and expiratory time (Ttot), (**c**) temperature behind the mask (Tmask), and (**d**) relative humidity behind the mask (RH) in 40 subjects wearing no mask (blue), surgical mask (yellow), community mask (green), and FFP2 (red).

During the 4-h workplace measurement, a slightly increased CO_2_ concentration behind the mask was detected (Fig. 4a). A small further increase was observed within the four hours, which did not lead to an increase in blood pCO_2_ or a decrease in pO_2_ and sO_2_. Under mask-wearing experimental conditions, inspiratory and expiratory time were longer than without mask (Fig. 4b). Increased temperature and relative humidity were measured behind the masks, but did not increase any further over the 4-h module period (Fig. 4c, d).

### Subjects’ perceived physical exertion

During CPET and ergometry, study subjects reported progressively higher BORG ratings during exercise when wearing a mask. For FFP2 these findings were statistically significant (Table 4). The differences in the BORG scale compared to the NM situation were also greater when the subjects wore masks for four hours at light work. Differences in the BORG scale compared to the NM situation were 0.67 (*p* = 0.278) for SM, 0.52 (*p* = 0.876) for CM, and 1.13 (*p* = 0.029) for FFP2.

## Discussion

### Principal findings

In this partially double-blinded randomized cross-over study wearing SM, CM, and FFP2 caused an increase in total airway resistance and specific airway resistance, which was most pronounced for FFP2. The increased breathing resistance led to an increase in work of breathing and respiratory power and thus to a slightly prolonged Ttot. These changes in breathing patterns were observed already at rest and were most pronounced with increasing workload during exercise.

With the increasing workload, a decrease was observed in sO_2_ and pO_2_ as well as an increase in pCO_2_. These changes normalized quickly when the physical exertion ended, even if wearing a mask was continued. No changes were measured in blood gas concentrations during 4-h at light to moderate workload (Module 4), whereas temperature and humidity behind the masks increased significantly.

## Strengths of the study

A special feature of our study is the complex study design which allowed to examine all 40 subjects in all four modules, partly even double-blinded, without and when wearing three different masks at different workloads and up to four hours continuously. Mask types were previously checked according to EN 149 and confirmed as representative mask models, especially according to the resistance of the filter material.

The study participants covered a wide a range of age, fitness, and some common diseases. Subjects in other studies were younger and, in general, only well-trained subjects were examined^14,20–22^. The PWC_130_ in our study was similar for both sex, reflecting moderate to well-trained subjects^36^. Exercise tests were performed with 6-min exercise levels to assure a physiological "steady state", which is a more precise verification of constant exercise thresholds (e.g. PWC_130/150_) than extrapolation from a ramp load^37^. The level of exercise was individually based on the respiratory minute volume for work-related stress, compared to previous studies that focused on recreational sport activities with sometimes very high workloads^14,20–22^. As a result, the biological variability of our measurement data was reduced.

The housing of a common bacterial filter was used as an adapter for the mask material to prevent influencing the ventilation measurement by the CPET silicone mask, which has been suspected as an influencing factor in studies where face masks were worn underneath the CPET mask^10,13^. In addition, the examinations could be carried out in a double-blind setting for the first time.

To prevent a systematic error due to one of the four different situations and to consider repeated measurements, an intraindividual comparison at the different examination times (i.e., each subject is compared to him/herself) was chosen. Due to the complex study design, we were able to compare the four different modules with each other and to link them by means of correlation analyses.

We have placed special emphasis on a sufficient washout period between the modules or the wearing of the different masks which was previously critically discussed by several papers^9,10^. This ensured that (adaptive) metabolic / physiological changes due to the preceding examination phase would not have any influence on the next examination phase and was proven by repeated baseline measurements (on exercise related parameters such as lactate or glucose for example) before the respective examinations. Furthermore, the different modules were processed in a randomized order to avoid any bias or residual effects of the former intervention.

## Limitations of the study

Due to the fact that exercise tests were carried out in air-conditioned laboratory rooms, effects of differing environmental conditions (e.g. temperature, humidity) on physiological parameters and the microclimate behind the mask could not be considered. Although some subjects with mild asthma and high blood pressure took part in the study, no statements can be made about subjects with more severe chronic lung or heart diseases^38^.

### Breathing physiology and cardiopulmonary data

#### Body plethysmography and CPET (Modules 1–2)

The increased breathing resistance due to the different filter materials of the face masks was confirmed by the testing on the Sheffield head according to EN 149. Body plethysmographic examinations showed increases in total airway resistance (Rtot), specific airway resistance, work of breathing and respiratory power, and slight decreases in FVC and FEV_1_. Interestingly, our findings do not match with previously published studies, possibly due to the wearing of face masks under the silicone CPET mask instead of using a mask adapter like in the present study. Some authors found a reduction in static and dynamic lung function parameters of nearly 30%^20,21^ and respiratory resistance was nearly twice as high with SM compared to no mask^13^. Wearing a mask behind a silicone mask obviously adds additional external resistance due to the tight and close fitting, which should be considered regarding the results of previously published studies. Shaw and co-authors^12^ also discussed that assessment of ventilation using a silicone CPET mask requires that the mask forms an adequate seal with the skin surface of the face to prevent air from escaping. When placing a silicone CPET mask before a face mask, this seal can be interrupted and negatively influence the measurements.

Engeroff and co-authors^9^ reported not only changes in respiratory rate and depth of breathing by wearing masks but also a trend to increased sO_2_ at rest (FFP2, SM). The authors explained these effects by hyperventilation. In contrast, our results showed a trend towards hypoventilation (longer inspiratory and expiratory time, lower minute ventilation) under resting conditions, resulting in a slight increase of pCO_2_ and decrease of pO_2_ and sO_2_. Wearing masks changes the breathing mechanic under resting conditions and subjects had to overcome a higher breathing resistance with the mask, making a single breath more strenuous.

Previous studies investigated the potential effects of masks using different exercise protocols and outcomes^14,17,20,22^, partly using a ramp protocol until maximum exertion^20,22^ or different load levels under ergometric (no CPET) exercise^17,21^.

During increased physical exercise, the human organism reacts with physiological adaptation (e.g., increased minute ventilation, heart rate), which can be measured precisely using CPET. In our study, the increase in minute ventilation was smaller wearing a mask compared to NM at each load level. This can be explained by the increased breathing resistance and an alteration in the breathing pattern due to the specific type of mask^9–12^. The breathing patterns in our study were changed due to an increased time of a single breath, thus a longer period is available in the pulmonary alveoli for the exchange of O_2_ and CO_2_. This conclusion is supported by a lower VE/VO_2_ and VE/VCO_2_ resulting in an improvement in breathing efficiency and a lower oxygen uptake (VO_2_) with increasing load while wearing a mask. This observation agrees with published data^9,14,20,22^.

The CPET examination findings regarding the improved breathing efficiency can be verified with the help of ergometric and workplace studies (modules 3 and 4) where the mask was worn under everyday use conditions. In these examinations a longer respiratory cycle time depending on the mask type (SM < CM < FFP2) was shown.

In addition, our data reveal further results concerning the effects of wearing a mask on breathing efficiency. Firstly, subjects with greater FVC and FEV_1_ had an increased inspiratory and expiratory time and therefore a better breathing efficiency (lower VE/VO_2_ and VE/VCO_2_), which was more pronounced when wearing a mask. This mechanism is primarily dependent on body size and sex and on the degree of a possible lung disease which may also affect FVC and FEV_1_. The advantage of an inspiratory and expiratory time (Ttot) resulting in a better breathing efficiency was, on the other hand, accompanied by increased specific airway resistance, work of breathing and pCO_2_ and lower pO_2_ and sO_2_. Although these relations have been suspected in previous studies^9,14,20,22^, our study is the first showing these relationships using correlation analysis.

Secondarily, based on the positive association between FVC, FEV_1_ and Ttot, it can be assumed that small individuals (children) or patients with lung disease are not able to increase Ttot sufficiently. Thus, the risk of having abnormal blood gas values as in some individuals in our study seems to be very low. These assumptions are supported by the fact that both small individuals (children, adolescents, small adults) and patients with pulmonary disease have increased specific airway resistance and work of breathing^39^. The increase in VE under exercise in these groups occurs primarily via an increase in respiratory rate due to hyperventilation, which can lead to a decrease in Ttot and to an inefficient breathing pattern with a decrease in pCO_2_^39–41^. However, these assumptions should be carefully examined in specific populations such as children or even small adults and in patients in further studies.

#### Ergometry (Module 3)

Presumably due to the physiological adaptation under CPET, a decrease of pO_2_ and sO_2_ and increase of pCO_2_ was also measured during ergometry. In individual cases the values decreased to the lower physiological limits. Inspiratory and expiratory time increased in the ergometry, too. It can be assumed that the changes of blood gases are caused by the effort for efficient breathing. The assumption is supported by the fact that both the subjects and the external load were identical during CPET and ergometry.

During physical exertion (up to approx. 150 watts and comparable to the PWC_130/150_), the partial rebreathing of the increased CO_2_ behind the mask led to significant blood gas changes consistent with the results of other studies^17,22^. Different results of other authors may be due to the use of shorter ramp protocols without a steady state^20–22^, FFP2 with exhalation valves^21^, wearing mask under the silicone CPET mask^14,20,22^, or the lack of verification by body plethysmography, CPET, and ergometry within one study group^17,20–22^.

#### Workplace (Module 4)

The small partial rebreathing of CO_2_ during light work for four hours (every-day use mask wearing condition) did not lead to changes in sO_2_, pO_2_ and pCO_2_ in our study. In a study conducted before the Corona pandemic, wearing SM for 30 min led to an accumulation of CO_2_ behind the mask and a significant increase of transcutaneous pCO_2_^40^. It is possible that the longer wearing time in our study (240 min) lead to a compensation (adaptation of respiration or acid–base balance), resulting in no significant difference in blood pCO_2_ values compared to studies with shorter wearing time^40,41^. In addition, the direct determination of CO_2_ in capillary blood is a more accurate method than a transcutaneous measurement, which estimates the pCO_2_ in arterial blood. However, in accordance with our results both studies^40,41^ did not observe a drop in sO_2_ when wearing masks during light work for a longer period.

Due to reduced permeability of the masks, the warm and humid exhaled breath condenses behind the mask and leads to increases in humidity and temperature. In addition to the breathing resistance of the mask, the material (cotton vs. synthetic) needs to be considered. Although testing on the Sheffield head showed for SM and CM a similar breathing resistance that was lower than that of FFP2, humidity was highest behind the CM during both, exercise and prolonged mask wear, probably due to the higher moisture absorption of cotton (CM) than of the synthetic material (SM).

The subjects' perceived physical exertion, especially when wearing the mask for a long time, was probably also influenced by the microclimate, primarily in terms of increased warmth and humidity behind the mask. Similar effects were seen in other studies^14,34,41^.

## Conclusions

In conclusion, wearing face masks caused significant physiological strain, but did not represent a health risk. Study participants reported a higher perceived physical exertion due to enhanced breathing resistance together with increased humidity and temperature behind the mask. All effects were most pronounced when wearing FFP2.

## Supplementary Information

Supplementary Information.

## Data Availability

Additional data will be supplied on reasonable request. The request should be addressed to the corresponding author.

## References

[1] Bagheri MH (2021). Filtration efficiency, breathability, and reusability of improvised materials for face masks. Aerosol Sci. Technol..

[2] Li Y (2021). Face masks to prevent transmission of COVID-19: A systematic review and meta-analysis. Am. J. Infect. Control.

[3] Ueki H (2020). Effectiveness of face masks in preventing airborne transmission of SARS-CoV-2. MSphere.

[4] Chu DK (2020). Physical distancing, face masks, and eye protection to prevent person-to-person transmission of SARS-CoV-2 and COVID-19: A systematic review and meta-analysis. Lancet.

[5] Ju JTJ, Boisvert LN, Zuo YY (2021). Face masks against COVID-19: Standards, efficacy, testing and decontamination methods. Adv. Colloid Interface Sci..

[6] Li Y (2021). Wearing masks to reduce the spread of respiratory viruses: a systematic evidence mapping. Ann. Transl. Med..

[7] Zhang M, Emery AR, Tannyhill RJ, Zheng H, Wang J (2020). Masks or N95 respirators during COVID-19 pandemic–which one should I wear?. J. Oral Maxillofac. Surg..

[8] Bünger J, Marek EM, van Kampen V, Bruening T (2022). Efficiency and burdens of wearing masks for protection against Sars-Cov-2: A narrative review focused on the current situation at workplaces. Res. Rev. Health Care Open Acc..

[9] Engeroff T, Groneberg DA, Niederer D (2021). The impact of ubiquitous face masks and filtering face piece application during rest, work and exercise on gas exchange, pulmonary function and physical performance: A systematic review with meta-analysis. Sports Med. Open.

[10] Grimm K, Niederer D, Engeroff T (2022). Blood gas levels, cardiovascular strain and cognitive performance during surgical mask and filtering face piece application. Sci. Rep..

[11] Hopkins SR (2021). Face masks and the cardiorespiratory response to physical activity in health and disease. Ann. Am. Thorac. Soc..

[12] Shaw KA (2021). The impact of face masks on performance and physiological outcomes during exercise: A systematic review and meta-analysis. Appl. Physiol. Nutr. Metab..

[13] Hopkins SR, Stickland MK, Schoene RB, Swenson ER, Luks AM (2020). Effects of surgical and FFP2/N95 face masks on cardiopulmonary exercise capacity: The numbers do not add up. Clin. Res. Cardiol..

[14] Lässing J (2020). Effects of surgical face masks on cardiopulmonary parameters during steady state exercise. Sci. Rep..

[15] Cabanillas-Barea S (2021). Effects of using the surgical mask and FFP2 during the 6-min walking test. A randomized controlled trial. Int. J. Environ. Res. Public Health.

[16] Doherty CJ (2021). Impact of wearing a surgical and cloth mask during cycle exercise. Appl. Physiol. Nutr. Metab..

[17] Georgi C, Haase-Fielitz A, Meretz D, Gaesert L, Butter C (2020). The impact of commonly-worn face masks on physiological parameters and on discomfort during standard work-related physical effort. Dtsch. Arztebl. Int..

[18] Reychler G, van der Straeten C, Schalkwijk A, Poncin W (2021). Effects of surgical and cloth facemasks during a submaximal exercise test in healthy adults. Resp. Med..

[19] Shein SL (2021). The effects of wearing facemasks on oxygenation and ventilation at rest and during physical activity. PLoS ONE.

[20] Fikenzer S (2020). Effects of surgical and FFP2/N95 face masks on cardiopulmonary exercise capacity. Clin. Res. Cardiol..

[21] Steinhilber B (2022). Effects of face masks on physical performance and physiological response during a submaximal bicycle ergometer test. Int. J. Environ. Res. Public Health.

[22] Mapelli M (2021). “You can leave your mask on": Effects on cardiopulmonary parameters of different airway protective. Eur. Respir. J..

[23] American Thoracic Society. American College of Chest Physicians (2003). ATS/ACCP statement on cardiopulmonary exercise testing. Am. J. Respir. Crit. Care Med..

[24] Palange, P., Laveneziana, P., Neder, J. & Ward S. A. (eds.). *Clinical Exercise Testing (ERS Monograph)* (European Respiratory Society, Sheffield) (2018).

[25] German Social Accident Insurance. DGUV Regel 112–190 Benutzung von Atemschutzgeräten; Deutsche Gesetzliche Unfallversicherung e.V. (DGUV): Berlin, Germany, 2021; Volume November 2021. https://www.google.com/url?sa=t&rct=j&q=&esrc=s&source=web&cd=&ved=2ahUKEwjPp6Lyrbn2AhVoQ_EDHQuIC2sQFnoECAoQAQ&url=https%3A%2F%2Fpublikationen.dguv.de%2Fwidgets%2Fpdf%2Fdownload%2Farticle%2F1011&usg=AOvVaw03c3WRczLT1LceCtM7SQm5. Last Accessed Aug 2022.

[26] Åstrand PO, Rodahl K, Dahl H, Dahl HA, Strømme SB (2003). Textbook of Work Physiology: Physiological Bases of Exercise.

[27] Jesus JP (2022). Effects of surgical masks on the responses to constant work-rate cycling performed at different intensity domains. Clin. Physiol. Funct. Imaging.

[28] Marek EM, Bode-Becker A, Volke J, Mueckenhoff K, Marek W (2012). Is the model of extrapolation of respiratory and circulatory parameters suitable for supporting measured resting values in ergometric exercise tests?. Pneumologie.

[29] EN 149:2001+A1:2009 Respiratory protective devices—Filtering half masks to protect against particles—Requirements, testing, marking; European Committee for Standardization: Brussels, Belgium (2009).

[30] Wanger J (2005). Standardisation of the measurement of lung volumes. Eur. Respir. J..

[31] Miller MR (2005). Standardisation of spirometry. Eur. Respir. J..

[32] D'Mello J, Butani M (2002). Capnography. Indian J. Anaesth..

[33] Yang Q (2018). Study of the micro-climate and bacterial distribution in the dead space of N95 filtering face respirators. Sci. Rep..

[34] Yoshihara A (2021). Effects of face mask use on objective and subjective measures of thermoregulation during exercise in the heat. Sports Health.

[35] Borg G (1998). Borg’s Perceived Exertion and Pain Scale.

[36] Finger JD, Krug S, Goeßwald A, Haertel S, Boes K (2013). Cardiorespiratory fitness among adults in Germany. Results of the German Health Interview and Examination Survey for Adults (DEGS1). Bundesgesundheitsblatt-Gesundheitsforschung-Gesundheitsschutz.

[37] Demarle AP (2021). Decrease of O_2_ deficit is a potential factor in increased time to exhaustion after specific endurance training. J. Appl. Physiol..

[38] Dellweg D (2020). Position paper of the German Respiratory Society (DGP) on the impact of community masks on self-protection and protection of others in regard to aerogen transmitted diseases. Pneumologie.

[39] Kyung SY, Kim Y, Hwang H, Park J-W, Jeong SH (2020). Risks of N95 face mask use in subjects with COPD. Respir. Care.

[40] Butz, U. Rebreathing of carbon dioxide of surgical staff using hygienic masks. Doctoral thesis. Technische Universität München (2005). http://nbn-resolving.de/urn/resolver.pl?urn:nbn:de:bvb:91-diss20050713-2027575920

[41] Sukul P (2022). Effects of COVID-19 protective face-masks and wearing durations onto respiratory-haemodynamic physiology and exhaled breath constituents. Eur. Respir. J..

