# Supplementary material for: Effects of wearing different face masks on cardiopulmonary performance at rest and exercise in a partially double-blinded randomized cross-over study
Source: Sci Rep. 2023 Apr 28;13:6950. doi: 10.1038/s41598-023-32180-9 (PMC10141827; doi:10.1038/s41598-023-32180-9)
Supplement: Supplementary file 1 — Supplementary Information. [file 41598_2023_32180_MOESM1_ESM.docx]

**Supplementary Data:**

**Effects of wearing face masks for protection against SARS-CoV-2 on cardiopulmonary performance in a** **partly double-blinded randomized cross-over study**

Eike-Maximillian Marek, Vera van Kampen, Birger Jettkant, Benjamin Kendzia, Bianca Strauß, Kirsten Sucker, Melanie Ulbrich, Anja Deckert, Hans Berresheim, Christian Eisenhawer, Frank Hoffmeyer, Simon Weidhaas, Thomas Behrens, Thomas Brüning, Jürgen Bünger

Table S1: Results (median (range)) of testing the different mask types for breathing resistance and filter efficiency according to EN 149.

|  | Breathing resistance according to EN 149  [Pa] Median (Range) | | | Filter efficiency according to EN 149 (Permeability for NaCl aerosol after 3 min)  [%] Median (Range) |
| --- | --- | --- | --- | --- |
| Mask type | at airflow 30 L/min^(I)^ | at airflow 95 L/min^(I)^ | at airflow 160 L/min^(E)^ |  |
| SM | 17 (12 - 18) | 93 (87 - 148) | 71 (56 - 77) | 33 (12 - 52) |
| CM | 16 (14 -19) | 101 (88 - 116) | 70 (58 - 80) | 65 (57 - 85) |
| FFP2 | 33 (30 - 35) | 143 (133 - 159) | 154 (146 - 166) | 0.04 (0.02 – 0.07) |

SM: surgical mask; CM: community mask; FFP2: filtering face piece class 2 ^(I)^: inspiratory resistance; ^(E)^: expiratory resistance

EN 149 is the European Standard that specifies requirements for FFP.
The maximum permitted breathing resistance for FFP2 is ≤ 70 Pa (30 L/min), ≤ 240 Pa (95 L/min), ≤ 300 Pa (160 L/min).

Table S2: CPET results of generalized linear mixed model (GLM model) analysis of 40 subjects without mask (NM) and with three different mask types (SM, CM, FFP2). Masks were tested using the mask adapter (details see Methods). The situation without mask in each specific load level (pre, E1, E2, E3, post) was always used as reference. For NM geometric mean (G.M.), for the three mask types (SM, CM, FFP2) differences (Δ) to NM and p-values are shown.

|  | Pre (light work) | | | | E1 (moderate work) | | | | E2 (heavy work) | | | | E3 (very heavy work) | | | | Post (light work | | | |
| --- | --- | --- | --- | --- | --- | --- | --- | --- | --- | --- | --- | --- | --- | --- | --- | --- | --- | --- | --- | --- |
|  | NM | SM | CM | FFP2 | NM | SM | CM | FFP2 | NM | SM | CM | FFP2 | NM | SM | CM | FFP2 | NM | SM | CM | FFP2 |
|  | G.M. | Δ | | | G.M. | Δ  p-value | | | G.M. | Δ  p-value | | | G.M. | Δ  p-value | | | G.M. | Δ  p-value | | |
| Subjects' perceived physical exertion | | | | | | | | | | | | | | | | | | | | |
| BORG  (0–10) | 0.69 | 0.19 | 0.03 | 0.23 | 1.38 | -0.05  0.140 | -0.04  0.732 | 0.55  0.780 | 2.81 | 0.00  0.174 | 0.10  0.992 | 0.79  0.802 | 4.47 | 0.21  0.269 | 0.41  0.774 | 1.11  0.685 | 1.05 | 0.25  0.882 | 0.19  0.539 | 0.35  0.966 |
| Hemodynamic parameters | | | | | | | | | | | | | | | | | | | | |
| HR  (min^-1^) | 82.70 | 1.47 | 0.37 | 0.98 | 104.62 | 1.83  0.992 | 0.16  0.899 | 1.53  0.902 | 127.52 | 1.83  0.882 | 0.64  0.980 | 1.62  0.965 | 150.53 | 1.29  0.667 | 1.07  0.902 | 1.31  0.884 | 106.36 | -0.62  0.300 | -1.34  0.448 | 1.57  0.894 |
| SBP  (mmHg) | 114.37 | -6.74 | -5.10 | 4.09 | 133.27 | 0.62  0.189 | -1.92  0.524 | -5.36  0.123 | 154.46 | -4.86  0.531 | -4.42  0.712 | -4.01  0.179 | 165.63 | 3.69  0.061 | -0.63  0.331 | 1.21  0.523 | 138.19 | 6.21  **0.038** | 3.05  0.172 | 5.20  0.972 |
| DBP  (mmHg) | 80.65 | -1.28 | 0.12 | -1.75 | 76.06 | -0.92  0.931 | -2.43  0.440 | 0.04  0.608 | 76.35 | -0.30  0.783 | -0.71  0.801 | 3.40  0.129 | 78.58 | -0.0  0.749 | 3.11  0.444 | 2.42  0.288 | 75.27 | 0.48  0.650 | 1.16  0.778 | 5.02  0.079 |
| Capnometric parameters | | | | | | | | | | | | | | | | | | | | |
| pCO_2_in (mmHg) | 7.86 | -1.28 | 0.42 | 0.32 | 6.85 | -0.97  0.847 | -0.28  0.478 | -0.65  0.297 | 6.89 | -1.08  0.954 | -0.21  0.531 | -0.66  0.298 | 6.87 | -1.55  0.569 | -0.60  0.288 | -0.84  0.213 | 8.03 | -1.50  0.850 | -0.39  0.491 | -0.91  0.280 |
| pCO_2_ex (mmHg) | 28.52 | 0.60 | 0.95 | -0.51 | 30.58 | 0.77  0.917 | 0.97  0.961 | 0.32  0.442 | 31.20 | 0.06  0.648 | -0.47  0.238 | -1.00  0.718 | 29.24 | -0.08  0.556 | 0.07  0.440 | 1.11  0.178 | 26.70 | 0.25  0.746 | 0.42  0.615 | 0.35  0.385 |
| Mask microclimate | | | | | | | | | | | | | | | | | | | | |
| Tmask (°) | 33.9 | -0.1 | -0.1 | -0.4 | 33.9 | -0.0  0.618 | 0.0  0.484 | -0.2  0.547 | 33.4 | 0.0  0.540 | 0.1  0.295 | -0.2  0.443 | 33.1 | 0.0  0.535 | 0.0  0.439 | -0.2  0.367 | 33.6 | -0.1  0.959 | -0.0  0.561 | -0.2  0.499 |
| RH (%) | 72.6 | -0.2 | 1.4 | 2.5 | 71.8 | -0.2  0.995 | 2.9  0.397 | 3.2  0.679 | 72.4 | 0.4  0.750 | 2.1  0.698 | 3.2  0.693 | 75.8 | 0.9  0.545 | 1.9  0.773 | 3.8  0.534 | 79.7 | 1.0  0.532 | 1.3  0.915 | 2.2  0.805 |

BORG: Borg scale; BR: Breathing reserve; CM: community mask; CPET: cardiopulmonary exercise test (spiroergometry); Δ: difference to NM; FFP2: filtering face piece class 2; G.M.: Geometric mean; NM: no mask; pCO_2_: partial pressure of carbon dioxide; pO_2_: partial pressure of oxygen; pH: potential of hydrogen in blood; RR: Respiratory rate; sO_2_: haemoglobin oxygen saturation; SM: surgical mask; Tin: inspiratory time; Tex: expiratory time; Ttot: inspiratory and expiratory time; VCO_2_: carbon dioxide production; VE: minute ventilation; VE/VCO_2_: ventilation per litre of carbon dioxide; VE/VO_2_: ventilation per litre of oxygen; VO_2_: oxygen uptake; VT: Tidal volume.

Table S3: CPET results (median, range) of 40 subjects without mask (NM) and with three different mask types (SM, CM, FFP2) for each specific load level (pre, E1, E2, E3, post).

|  | Pre (light work) | | | | E1 (moderate work) | | | | E2 (heavy work) | | | | E3 (very heavy work) | | | | Post (light work) | | | |
| --- | --- | --- | --- | --- | --- | --- | --- | --- | --- | --- | --- | --- | --- | --- | --- | --- | --- | --- | --- | --- |
|  | NM | SM | CM | FFP2 | NM | SM | CM | FFP2 | NM | SM | CM | FFP2 | NM | SM | CM | FFP2 | NM | SM | CM | FFP2 |
|  | median  range | | | | median  range | | | | median  range | | | | median  range | | | | median  range | | | |
| Performance parameters | | | | | | | | | | | | | | | | | | | | |
| Workload  (Watt) |  |  |  |  | 55 45-70 | 55 45-70 | 55 45-70 | 55 45-70 | 90 65-135 | 90 65-135 | 90 65-135 | 90 65-135 | 130 95-175 | 130 95-175 | 130 95-175 | 130 95-175 |  |  |  |  |
| Subjects' perceived physical exertion | | | | | | | | | | | | | | | | | | | | |
| BORG  (0–10) | 0 0-2 | 0 0-3 | 0 0-3 | 0.5 0-3 | 1 0.5-4 | 1 0.5-4 | 1 0-3 | 2 1-4 | 3 1-6 | 3 1-6 | 3 1-5 | 4 2-8 | 5 2-8 | 5 2-9 | 5 2-9 | 6 3-10 | 1 0-5 | 1 0-5 | 1 0-3 | 2 0-4 |
| Pulmonary parameters | | | | | | | | | | | | | | | | | | | | |
| Ttot  (s) | 3.86 2.5-5.8 | 3.84  2.4-5.5 | 3.90  2.7-6.1 | 4.08  2.7-6.6 | 2.86  1.8-4.4 | 2.95  1.9-4.6 | 2.87  1.8-4.7 | 3.15  1.9-5.3 | 2.56  1.5-3.9 | 2.58  1.4-4.8 | 2.47  1.5-3.8 | 2.64  1.6-4.8 | 2.09  1.3-3.3 | 2.08  1.2-3.5 | 2.15  1.3-3.3 | 2.33  1.5-3.5 | 2.96  1.7-5.7 | 2.93  1.9-4.6 | 2.94  1.6-4.0 | 3.09  1.9-5.0 |
| RR  (min^-1^) | 16 11-24 | 16 6-25 | 15 10-22 | 15 10-22 | 21 14-34 | 20 13-32 | 21 13-34 | 19 11-31 | 24 16-39 | 23 13-42 | 24 16-40 | 23 12-39 | 29 18-48 | 29 17-49 | 28 18-47 | 26 17-41 | 21 4-37 | 20 13-31 | 20 15-37 | 20 12-32 |
| VT  (L) | 0.88 0.6-1.6 | 0.89 0.5-2.0 | 0.90 0.5-1.5 | 0.87 0.6-1.5 | 1.65 1.1-2.3 | 1.65 1.1-2.6 | 1.63 1.1-2.4 | 1.64 1.2-3.1 | 1.95 1.3-2.9 | 1.83 1.3-3.1 | 1.90 1.3-2.8 | 1.94 1.3-3.3 | 2.19 1.5-3.2 | 2.20 1.6-3.3 | 2.18 1.6-3.2 | 2.19 1.3-3.5 | 1.2 0.9-2.1 | 1.2 0.8-2.1 | 1.2 0.9-1.9 | 1.2 0.6-2.2 |
| VE (L*min^−1^) | 13 8-16 | 13 10-18 | 12 9-19 | 13 9-17 | 33 23-45 | 30 26-40 | 31 25-45 | 30 23-44 | 46 38-68 | 47 33-60 | 43 39-56 | 30 23-44 | 63 47-88 | 59 48-78 | 56 45-76 | 56 55-76 | 25 15-31 | 22 14-31 | 21 14-32 | 22 17-36 |
| VO_2_ (ml*min^−1^) | 316 219-510 | 283 203-442 | 282 150-552 | 289 181-357 | 1102 811-1300 | 1055 811-1300 | 1002 867-1478 | 982 767-1230 | 1527 1211-1822 | 1552 1187-1693 | 1465 1208-1736 | 982 767-1230 | 1963 1503-2317 | 1862 1484-2268 | 1870 1644-2227 | 1856 1484-2259 | 571 150-752 | 513 262-724 | 428 292-795 | 523 361-770 |
| VCO_2_ (ml*min^−1^) | 258 175-388 | 228 148-344 | 232 107-437 | 237 169-296 | 908 721-1163 | 867 678-1104 | 871 678-1309 | 834 649-1163 | 1458 1117-1619 | 1410 1090-1538 | 1416 1023-1603 | 833 649-1163 | 1861 1476-2273 | 1815 1452-2244 | 1820 1451-2254 | 1784 1451-2254 | 500 107-728 | 469 277-676 | 425 281-697 | 485 337-850 |
| VE/VO_2_ | 40.64 26-55 | 45.53 32-82 | 42.70 34-61 | 43.29 29-61 | 30.01 23-38 | 30.22 24-36 | 30.45 24-38 | 30.75 24-37 | 30.30 23-42 | 30.91 21-42 | 31.38 22-40 | 29.53 21-40 | 32.58 23-46 | 32.85 22-49 | 31.52 22-46 | 30.12 22-44 | 40.89 31-54 | 44.29 32-64 | 43.97 33-66 | 40.73 32-109 |
| VE/VCO_2_ | 50.00 35-68 | 54.06 42-104 | 52.12 41-84 | 52.91 42-74 | 34.95 29-45 | 36.39 28-45 | 35.62 30-46 | 35.44 29-44 | 34.01 27-45 | 33.97 24-42 | 32.92 27-44 | 32.63 24-43 | 33.22 25-47 | 33.77  23-45 | 33.28 25-46 | 32.22 23-44 | 44.51 34-65 | 47.43 33-65 | 46.61 37-65 | 44.24 33-90 |
| EELV (L) | 4.38 1.6-7.5 | 4.22 0-7.5 | 4.48 2.5-7.8 | 4.15 0.8-7.4 | 4.09 2.3-7.7 | 3.96 2.3-7.5 | 3.88 1.6-7.5 | 4.41 2.6-7.2 | 4.12 1.9-7.4 | 3.79 2.1-7.6 | 4.23 1.7-6.2 | 3.65 1.9-7.4 | 4.08 1.8-7.4 | 3.50 2.3-5.7 | 3.68 1.7-7.3 | 3.52 2.0-6.2 | 4.68 2.3-7.6 | 4.32 2.2-8.0 | 4.17 1.9-7.6 | 3.77 1.2-6.2 |
| BR (%) | 11.49 6-18 | 11.47 5-20 | 12.24 7-16 | 10.57 5-19 | 27.42 19-48 | 27.05 19-56 | 26.56 18-56 | 26.62 18-51 | 39.71 26-74 | 38.60 25-80 | 40.14 27-77 | 36.94 25-77 | 54.97 34-97 | 55.81 33-99 | 53.92 34-97 | 48.97 31-103 | 20.31 11-37 | 19.30 10-38 | 20.64 10-40 | 20.26 12-41 |
| Metabolic parameters | | | | | | | | | | | | | | | | | | | | |
| pH | 7.41 7.3-7.5 | 7.41  7.3-7.5 | 7.41  7.3-7.5 | 7.41  7.3-7.5 | 7.40  7.3-7.5 | 7.39  7.3-7.5 | 7.40  7.3-7.4 | 7.39  7.3-7.5 | 7.39  7.3-7.5 | 7.38  7.3-7.4 | 7.38  7.3-7.5 | 7.38  7.3-7.5 | 7.37  7.3-7.5 | 7.36  7.2-7.4 | 7.36  7.3-7.4 | 7.36  7.2-7.4 | 7.38 7.3-7.5 | 7.38  7.3-7.5 | 7.38  7.3-7.5 | 7.38  7.3-7.5 |
| pCO_2_ (mmHg) | 36.8  28-43 | 36.5  25-49 | 37.5  29-44 | 37.65  29-48 | 38.5  30-46 | 39.0  32-53 | 38.05  34-46 | 39.3  30-56 | 38.6  28-45 | 38.0  29-55 | 39.3  30-46 | 39.7  28-55 | 36.8  26-44 | 37.4  27-51 | 38.2  27-47 | 38.5  28-54 | 35.6  25-41 | 35.3  25-46 | 35.4  26-41 | 35.4  25-49 |
| pO_2_ (mmHg) | 88.7  78-114 | 87.5  62-111 | 90.4  69-110 | 88.4  70-101 | 92.1  81-108 | 89.3  74-103 | 91.6  79-104 | 89.2  76-108 | 91.0  70-105 | 87.5 71-110 | 89.8  71-103 | 88.3  74-104 | 90.4  65-105 | 85.9  65-105 | 87.7  61-102 | 87.3  67-109 | 94.0  79-111 | 93.1  80-109 | 95.1  80-110 | 95.7  81-108 |
| Lactate (mmol/L) | 1.2  0.7-2.3 | 1.2  0.7-3.3 | 1.2  0.6-3.1 | 1.3  0.6-2.1 | 1.5  0.7-4.2 | 1.4  0.7-4.2 | 1.6 0.7-4.2 | 1.7  0.9-3.5 | 2.9 1.0-5.0 | 2.6 0.8-5.9 | 2.7  0.8-7.1 | 2.5  1.1-5.3 | 5.0  2.0-9.4 | 4.6  1.4-9.2 | 5.0 1.8-9.9 | 4.9  2.2-12 | 4.1 1.2-8.1 | 3.8  1.1-8.7 | 4.0 1.2-9 | 4.1  1.5-10 |
| sO_2_  (%) | 97.4  96-98 | 97 .0 94- 98 | 97.1 95- 99 | 97.0 95- 98 | 97.4 96- 99 | 97.1 95-99 | 97.1  96-99 | 97.1  94- 99 | 97.0  94-98 | 96.8  94-98 | 97.0  94-98 | 96.9  94-98 | 96.9  94-100 | 96.5 95-98 | 96.7 93-98 | 96.5  94-98 | 97.2  95-99 | 97.2  95- 98 | 97.2 95-99 | 97.1  95-99 |
| Hemodynamic parameters | | | | | | | | | | | | | | | | | | | | |
| HR (min^-1^) | 87 57-107 | 89 63-104 | 81 62-100 | 89 60-109 | 108 78-137 | 103 78-131 | 105 78-122 | 107 76-136 | 127 92-164 | 131 89-160 | 131 95-148 | 132 91-164 | 153 112-184 | 155 110-184 | 151 113-181 | 157 114-179 | 108 74-136 | 106 75-133 | 103 71-126 | 108 85-137 |
| SBP (mmHg) | 116 94-135 | 112 88-126 | 113 78-133 | 116 98-164 | 130 106-171 | 139 86-165 | 129 113-160 | 129 86-169 | 148 129-193 | 145 115-198 | 153 110-193 | 151 105-196 | 163 145-225 | 163 145-225 | 163 115-224 | 166 134-221 | 136 107-196 | 140 123-186 | 144 103-200 | 140 116-185 |
| DBP (mmHg) | 81 63-96 | 81 53-93 | 84 61-107 | 83 53-99 | 75 58-94 | 75 54-90 | 70 62-95 | 76 53-105 | 76 61-100 | 74 61-102 | 75 55-100 | 80 60-114 | 80 54-96 | 80 54-96 | 85 58-103 | 80 60-137 | 78 57-93 | 80 53-98 | 78 59-93 | 80 60-122 |
| Capnometric parameters | | | | | | | | | | | | | | | | | | | | |
| pCO_2_in (mmHg) | 7.76 4.5-14 | 7.75 0.1-16 | 8.04 4.5-16 | 8.08 3.6-16 | 6.62 4.1-13 | 6.43 0.7-14 | 6.47 4.4-11 | 6.12 4.0-13 | 6.45 4.3-13 | 6.08 1.7-13 | 6.29 4.2-14 | 6.34 1.4-16 | 6.72 4.4-13 | 5.85 0.5-12 | 5.98 3.7-14 | 5.97 3.7-14 | 7.83 4.6-16 | 7.14 0.3-15 | 7.53 3.6-14 | 7.19 0.7-16 |
| pCO_2_ex (mmHg) | 29.47 21-37 | 30.80 18-40 | 30.41 21-35 | 18.64 17-38 | 31.88 21-38 | 33.25 16-41 | 32.77 18-37 | 32.39 18-39 | 32.00 22-40 | 32.91 15-41 | 32.70 15-38 | 32.39 11-41 | 29.04 20-42 | 30.66 15-39 | 31.09 12-39 | 31.11 21-40 | 27.43 19-36 | 29.26 14-36 | 28.77 18-35 | 27.99 18-35 |
| Mask microclimate | | | | | | | | | | | | | | | | | | | | |
| Tmask (°C) | 34.0 32-36 | 33.8 33-36 | 33.8 32-35 | 33.6 32-36 | 34.1 32-36 | 33.9 32-36 | 34.0 32-35 | 33.6 32-35 | 33.4 32-35 | 33.4 32-35 | 33.6 32-35 | 33.1 32-35 | 33.0 32-35 | 33.1 32-35 | 33.2 31-35 | 32.9 32-35 | 33.4 32-36 | 33.7 31-36 | 33.6 31.36 | 33.2 32-36 |
| RH (%) | 71.6 55-87 | 73.0 56-83 | 73.9 62-90 | 76.6 51-91 | 71.8 48-87 | 72.1 55-85 | 74.5 61-91 | 75.9 47-91 | 73.0 53-89 | 72.5 59-91 | 74.3 60-96 | 76.3 51-90 | 76.1 60-92 | 75.6 65-94 | 78.8 61-99 | 81.1 59-96 | 80.8 62-95 | 80.0 67-96 | 81.6 58-98 | 83.3 61-97 |

BORG: Borg scale; BR: Breathing reserve; CM: community mask; CPET: cardiopulmonary exercise test (spiroergometry); DBP: Diastolic blood pressure; EELV: end-expiratory lung volume; FFP2: filtering face piece class 2; HR: Heart rate; NM: no mask; pCO_2_: partial pressure of carbon dioxide; pO_2_: partial pressure of oxygen; pCO_2_ex: expiratory carbon dioxide pressure; pCO_2_in: inspiratory carbon dioxide pressure, pH: potential of hydrogen in blood; RH: relative humidity; RR: Respiratory rate; SBP: Systolic blood pressure; sO_2_: haemoglobin oxygen saturation; SM: surgical mask; Tmask: temperature behind the mask; Ttot: inspiratory and expiratory time; VCO_2_: carbon dioxide production; VE: minute ventilation; VE/VCO_2_: ventilation per litre of carbon dioxide; VE/VO_2_: ventilation per litre of oxygen; VO_2_: oxygen uptake; VT: Tidal volume.

Table S4: Correlations of body plethysmography and CPET results (Pearson Correlation (r) and 95% CI with Fisher transformation))

| NM | | CPET parameters | | | | | | | | | | | | | | | | |
| --- | --- | --- | --- | --- | --- | --- | --- | --- | --- | --- | --- | --- | --- | --- | --- | --- | --- | --- |
| Body plethysmography parameter |  | BORG | pCO_2_in | | Ttot | | VE | | VE/VO_2_ | | VE/VCO_2_ | | pCO_2_ | | pO_2_ | | sO_2_ | |
|  | FVC | -0.037  (-0.175;0.102) | 0.084  (-0.061;0.226) | | 0.228  (0.090;0.356) | | 0.014  (-0.127;0.154) | | -0.208  (-0.338;-0.068) | | -0.137  (-0.273;0.004) | | 0.111  (-0.029;0.245) | | 0.026  (-0.113;0.165) | | 0.181  (0.042;0.313) | |
|  | FEV_1_ | -0.011  (-0.150;0.128) | 0.056  (-0.090;0.198) | | 0.126  (-0.015;0.262) | | 0.011  (-0.130;0.151) | | -0.175  (-0.308;-0.035) | | -0.131  (-0.267;0.010) | | 0.158  (0.020;0.290) | | 0.060  (-0.080;0.197) | | 0.185  (0.045;0.316) | |
|  | Rtot | -0.063  (-0.200;0.077) | 0.019  (-0.126;0.163) | | -0.186  (-0.317;-0.046) | | 0.074  (-0.068;0.212) | | 0.167  (0.026;0.300) | | 0.111  (-0.031;0.247) | | -0.140  (-0.273;-0.001) | | 0.041  (-0.099;0.179) | | -0.189  (-0.321;-0.050) | |
|  | sRtot | -0.072  (-0.209;0.068) | 0.085  (-0.060;0.227) | | -0.011  (-0.151;0.130) | | 0.065  (-0.076;0.204) | | 0.005  (-0.136;0.145) | | 0.004  (-0.137;0.145) | | -0.026  (-0.164;0.114) | | 0.003  (-0.136;0.142) | | -0.159  (-0.292;-0.019) | |
|  | WOB | -0.045  (-0.183;0.094) | 0.170(0.025;  0.306) | | 0.092  (-0.049;0.229) | | 0.042  (-0.099;0.181) | | -0.026  (-0.166;0.115) | | -0.064  (-0.203;0.078) | | 0.0126  (-0.126;0.151) | | 0.011  (-0.129;0.149) | | -0.149  (-0.283;-0.009) | |
|  | RP | -0.002  (-0.141;0.137) | 0.130  (-0.015;0.269) | | 0.051  (-0.090;0.190) | | 0.057  (-0.084;0.196) | | 0.021  (-0.120;0.162) | | -0.015  (-0.156;0.126) | | -0.013  (-0.151;0.126) | | 0.012  (-0.127;0.151) | | -0.197  (-0.328;-0.058) | |
|  | Ttot | -0.101  (-0.237;0.039) | 0.019  (-0.126;0.163) | | 0.146  (0.005;0.280) | | -0.061  (-0.199;0.081) | | -0.110  (-0.247;0.032) | | -0.148  (-0.282;-0.006) | | 0.101  (-0.039;0.236) | | -0.112  (-0.247;0.028) | | 0.132  (-0.009;0.267) | |
| SM | | CPET parameters | | | | | | | | | | | | | | | | |
| Body plethysmography parameter |  | BORG | pCO_2_in | | Ttot | | VE | | VE/VO_2_ | | VE/VCO_2_ | | pCO_2_ | | pO_2_ | | sO_2_ | |
|  | FVC | -0.128  (-0.262;0.012) | -0.075  (-0.215;0.068) | | 0.255  (0.118;0.381) | | -0.026  (-0.166;0.115) | | -0.187  (-0.319;-0.048) | | -0.117  (-0.253;0.025) | | 0.113  (-0.026;0.248) | | 0.109  (-0.031;0.244) | | 0.250  (0.114;0.376) | |
|  | FEV_1_ | -0.103  (-0.239;0.036) | -0.012  (-0.154;0.131) | | 0.162  (0.022;0.295) | | -0.036  (-0.175;0.106) | | -0.159  (-0.292;-0.018) | | -0.104  (-0.241;0.037) | | 0.194  (0.057;0.324) | | 0.117  (-0.022;0.252) | | 0.216  (0.078;0.345) | |
|  | Rtot | -0.020  (-0.159;0.199) | 0.005  (-0.137;0.147) | | -0.027  (-0.167;0.114) | | 0.073  (-0.068;0.211) | | 0.078  (-0.064;0.216) | | 0.069  (-0.073;0.207) | | -0.251  (-0.276;-0.115) | | -0.063  (-0.199;0.077) | | -0.092  (-0.229;0.049) | |
|  | sRtot | -0.055  (-0.193;0.085) | -0.024  (-0.166;0.119) | | 0.202  (0.063;0.333) | | 0.039  (-0.102;0.179) | | -0.081  (-0.219;0.061) | | -0.051  (-0.190;0.090) | | -0.111  (-0.245;0.029) | | -0.081  (-0.217;0.059) | | 0.014  (-0.126;0.154) | |
|  | WOB | -0.113  (-0.248;0.027) | -0.047  (-0.188;0.096) | | 0.211  (0.072;0.341) | | 0.014  (-0.127;0.154) | | -0.130  (-0.265;0.011) | | -0.110  (-0.247;0.031) | | 0.079  (-0.061;0.215) | | 0.011  (-0.128;0.149) | | -0.029  (-0.168;0.111) | |
|  | RP | -0.090  (-0.226;0.050) | 0.059  (-0.084;0.200) | | 0.127  (-0.014;0.262) | | 0.028  (-0.113;0.168) | | -0.085  (-0.222;0.057) | | -0.067  (-0.206;0.074) | | -0.014  (-0.153;0.125) | | 0.098  (-0.042;0.233) | | 0.012  (-0.128;0.152) | |
|  | Ttot | -0.018  (-0.157;0.121) | -0.298  (-0.421;-0.161) | | 0.202  (0.063;0.333) | | -0.047  (-0.186;0.095) | | -0.098  (-0.235;0.043) | | -0.104  (-0.240;0.038) | | 0.304  (0.171;0.424) | | -0.367  (-0.481;-0.240) | | -0.066  (-0.203;0.075) | |
| CM | | CPET parameters | | | | | | | | | | | | | | | | |
| Body plethysmography parameter |  | BORG | | pCO_2_in | | Ttot | | VE | | VE/VO_2_ | | VE/VCO_2_ | | pCO_2_ | | pO_2_ | | sO_2_ |
|  | FVC | -0.099  (-0.234;0.041) | | 0.110  (-0.029;0.245) | | 0.198  (0.059;0.329) | | -0.005  (-0.145;0.136) | | -0.169  (-0.302;-0.029) | | -0.157  (-0.291;-0.017) | | 0.130  (-0.010;0.264) | | 0.047  (-0.093;0.185) | | 0.071  (-0.070;0.209) |
|  | FEV_1_ | -0.098  (-0.234;0.041) | | 0.072  (-0.068;0.208) | | 0.106  (-0.035;0.243) | | -0.016  (-0.156;0.125) | | -0.133  (-0.268;0.008) | | -0.138  (-0.273;0.002) | | 0.171  (0.032;0.302) | | 0.065  (-0.075;0.203) | | 0.026  (-0.115;0.165) |
|  | Rtot | -0.042  (-0.179;0.098) | | 0.205  (0.068;0.334) | | -0.036  (-0.175;0.105) | | 0.005  (-0.136;0.145) | | -0.005  (-0.145;0.136) | | 0.017  (-0.124;0.157) | | -0.143  (-0.276;-0.004) | | 0.084  (-0.056;0.221) | | -0.024  (-0.163;0.117) |
|  | sRtot | -0.094  (-0.229;0.046) | | 0.318  (0.187;0.437) | | 0.195  (0.056;0.326) | | -0.014  (-0.154;0.127) | | -0.168  (-0.301;-0.028; | | -0.114  (-0.250;0.027) | | -0.009  (-0.148;0.130) | | 0.016  (-0.124;0.155) | | 0.006  (-0.135;0.146) |
|  | WOB | -0.116  (-0.252;0.025) | | 0.195  (0.056;0.326) | | 0.160  (0.018;0.296) | | -0.033  (-0.174;0.110) | | -0.193  (-0.326;-0.051) | | -0.121  (-0.258;0.022) | | 0.042  (-0.100;0.181) | | 0.059  (-0.083;0.199) | | -0.010  (-0.152;0.132) |
|  | RP | -0.097  (-0.232;0.043) | | 0.226  (0.090;0.353) | | 0.198  (0.059;0.329) | | -0.028  (-0.168;0.113) | | -0.166  (-0.299;-0.026) | | -0.134  (-0.269;0.007) | | 0.109  (-0.031;0.244) | | 0.114  (-0.027;0.249) | | -0.055  (-0.193;0.086) |
|  | Ttot | -0.028  (-0.166;0.111) | | -0.081  (-0.217;0.059) | | 0.251  (0.114;0.278) | | -0.031  (-0.170;0.110) | | -0.126  (-0.262;0.015) | | -0.146  (-0.280;-0.005) | | 0.092  (-0.048;0.228) | | -0.328  (-0.446;-0.197) | | -0.045  (-0.184;0.096) |
| FFP2 | | CPET parameters | | | | | | | | | | | | | | | | |
| Body plethysmography parameter |  | BORG | pCO_2_in | | Ttot | | VE | | VE/VO_2_ | | VE/VCO_2_ | | pCO_2_ | | pO_2_ | | sO_2_ | |
|  | FVC | -0.141  (-0.275;-0.002) | -0.044  (-0.184;0.098) | | 0.328  (0.196;0.447) | | 0.002  (-0.138;0.143) | | -0.263  (-0.388;-0.126) | | -0.175  (-0.307;-0.034) | | 0.105  (-0.035;0.240) | | -0.022  (-0.160;0.118) | | 0.088  (-0.053;0.226) | |
|  | FEV_1_ | -0.129  (-0.263;0.011) | -0.062  (-0.201;0.080) | | 0.240  (0.102;0.367) | | 0.006  (-0.134;0.147) | | -0.236  (-0.364;-0.098) | | -0.156  (-0.289;-0.015) | | 0.103  (-0.037;0.238) | | -0.016  (-0.154;0.124) | | 0.130  (-0.011;0.266) | |
|  | Rto_t_ | -0.030  (-0.169;0.109) | -0.128  (-0.264;0.014) | | 0.044  (-0.097;0.184) | | -0.036  (-0.176;0.105) | | 0.057  (-0.085;0.195) | | 0.025  (-0.116;0.165) | | -0.062  (-0.199;0.078) | | -0.053  (-0.190;0.087) | | -0.044  (-0.183;0.097) | |
|  | sRtot | -0.045  (-0.182;0.095) | -0.140  (-0.275;0.002) | | 0.260  (0.123;0.386) | | -0.053  (-0.192;0.088) | | -0.092  (-0.229;0.050) | | -0.079  (-0.217;0.063) | | -0.013  (-0.152;0.126) | | -0.083  (-0.219;0.057) | | -0.015  (-0.155;0.126) | |
|  | WOB | -0.121  (-0.258;0.020) | 0.007  (-0.137;0.150) | | 0.277  (0.140;0.403) | | 0.012  (-0.131;0.154) | | -0.083  (-0.222;0.060) | | -0.069  (-0.209;0.075) | | -0.076  (-0.214;0.065) | | 0.099  (-0.043;0.237) | | 0.126  (-0.017;0.263) | |
|  | RP | -0.113  (-0.248;0.020) | -0.090  (-0.228;0.053) | | 0.262  (0.125;0.388) | | 0.024  (-0.117;0.164) | | -0.113  (-0.249; 0.028) | | -0.096  (-0.233;0.046) | | -0.073  (-0.210;0.066) | | 0.119  (-0.021;0.254) | | 0.033  (-0.108;0.173) | |
|  | Ttot | -0.031  (-0.169;0.109) | -0.248  (-0.375;-0.110) | | 0.252  (0.115;0.379) | | -0.124  (-0.259;0.018) | | -0.058  (-0.197;0.083) | | -0.134  (-0.269;0.007) | | 0.459  (0.341;0.561) | | -0.292  (-0.414;-0.159) | | -0.200  (-0.331;-0.061) | |

BORG: Borg scale; CM: community mask; FEV_1_: forced expiratory volume in 1 s; FFP2: filtering face piece class 2; FVC: forced vital capacity; NM: no mask; RP: respiratory power; Rtot: total airway resistance; WOB: work of breathing; pCO_2_in: inspiratory carbon dioxide pressure; pCO_2_: partial pressure of carbon dioxide; pO_2_: partial pressure of oxygen; SM: surgical mask; sO_2_: haemoglobin oxygen saturation; sRtot: specific airway resistance; Ttot: inspiratory and expiratory time; VE: minute ventilation; VE/VO_2_: ventilation per litre of oxygen; VE/VCO_2_: ventilation per litre of carbon dioxide.

Table S5: Ergometry results (median, range) of 40 subjects without mask (NM) and with three different mask types (SM, CM, FFP2) for each specific load level (pre, E1, E2, E3, post).

|  | Pre (light work) | | | | E1 (moderate work) | | | | E2 (heavy work) | | | | E3 (very heavy work) | | | | Post (light work) | | | |
| --- | --- | --- | --- | --- | --- | --- | --- | --- | --- | --- | --- | --- | --- | --- | --- | --- | --- | --- | --- | --- |
|  | NM | SM | CM | FFP2 | NM | SM | CM | FFP2 | NM | SM | CM | FFP2 | NM | SM | CM | FFP2 | NM | SM | CM | FFP2 |
|  | median  range | | | | median  range | | | | median  range | | | | median  range | | | | median  range | | | |
| Performance parameters | | | | | | | | | | | | | | | | | | | | |
| Workload  (Watt) |  |  |  |  | 55 45-70 | 55 45-70 | 55 45-70 | 55 45-70 | 90 65-135 | 90 65-135 | 90 65-135 | 90 65-135 | 130 95-175 | 130 95-175 | 130 95-175 | 130 95-175 |  |  |  |  |
| Subjects' perceived physical exertion | | | | | | | | | | | | | | | | | | | | |
| BORG  (0–10) | 0 0-1 | 0 0-2 | 0 0-1 | 0 0-2 | 1 0-3 | 1 0-3 | 1 0-3 | 1 0.5-4 | 2 0.5-4 | 3 1-5 | 3 0.5-5 | 3 1-5 | 4 1-7 | 4 2-9 | 4 2-9 | 5 2-9 | 0.5 0-3 | 1 0-3 | 1 0-3 | 1 0-5 |
| Pulmonary parameters | | | | | | | | | | | | | | | | | | | | |
| RR (min^-1^) | 19 13-27 | 19 12-30 | 19 12-32 | 18 10-27 | 24 14-42 | 24 13-49 | 22 15-49 | 22 14-39 | 27 16-49 | 26 15-49 | 24 17-42 | 27 17-42 | 30 19-45 | 30 19-49 | 30 19-45 | 30 19-45 | 22 16-48 | 22 17-35 | 24 17-45 | 23 16-49 |
| Ttot  (s) | 2.84 2.1-4.8 | 3.26 2.2-4.7 | 3.27 2.1-4.8 | 3.6 2.0-5.2 | 2.46 1.7-3.9 | 2.57 1.7-3.6 | 2.58 1.8-4.1 | 2.65 1.7-4.2 | 2.28 1.6-3.3 | 2.33 1.5-3.6 | 2.30 1.6-3.6 | 2.23 1.4-3.5 | 1.96 1.2-3.1 | 2.00 1.3-3.1 | 2.01 1.3-3.1 | 1.91 1.4-3.1 | 2.40 1.8-3.5 | 2.55 1.7-3.5 | 2.55 1.7-3.8 | 2.60 1.7-3.8 |
| Metabolic parameters | | | | | | | | | | | | | | | | | | | | |
| pH | 7.41  7.3-7.5 | 7.39  7.3-7.5 | 7.39 7.3-7.5 | 7.38 7.3-7.5 | 7.41  7.3-7.5 | 7.39 7.3-7.4 | 7.40  7.3-7.5 | 7.39  7.3-7.5 | 7.40 7.3-7.5 | 7.38 7.3-7.5 | 7.39 7.3-7.5 | 7.37 7.3-7.5 | 7.39 7.3-7.5 | 7.36 7.3-7.4 | 7.36 7.3-7.5 | 7.35 7.2-7.4 | 7.40  7.3-7.5 | 7.39 7.3-7.5 | 7.38 7.3-7.5 | 7.38  7.3-7.5 |
| pCO_2_ (mmHg) | 36.8 27-43 | 37.3  28-46 | 36.7 27-42 | 36.7  29-45 | 36.8  29-43 | 38.5 32-43 | 38.2  29-46 | 39.7  30-45 | 36.3  28-43 | 38.8  31-46 | 39.0  29-46 | 40.5  30-46 | 34.5  25-45 | 37.6  27-45 | 37.8  26-46 | 39.8  31-49 | 34.1 24-41 | 35.5  24-40 | 34.3  25-40 | 35.9  26-42 |
| pO_2_ (mmHg) | 89.1  66-104 | 91.0  71-106 | 89.3  72-108 | 88.6  70-107 | 92.1  78-105 | 90.3 73-104 | 93.4 79-105 | 90.6 71- 103 | 92.2 77-113 | 91.4  71-109 | 89.4 77-100 | 88.1 68-104 | 91.4  74-111 | 88.0  65-105 | 87.0  68-100 | 88.3 65-101 | 94.0  81-108 | 96.6  72-109 | 95.7 83-107 | 97.3  80-107 |
| Lactate (mmol/L) | 1.3  0.7-3.6 | 1.4  0.7-3.2 | 1.2  0.7-2.7 | 1.4  0.7-2.9 | 1.6  0.8-4.6 | 1.5  0.6-4.7 | 1.7  0.7-4 | 1.5  0.6-3.8 | 2.6  0.8-6.9 | 2.6  0.7-6.2 | 2.6  0.8-6.1 | 2.3  0.7-6.4 | 5.1  1.3-9.3 | 5  1.1-8.5 | 4.7  1.2-8.1 | 4.9  1.2-9.4 | 3.6  0.8-9.0 | 3.8  0.8-8.5 | 4.0  1.0-8.0 | 3.9  0.9-10 |
| sO_2_  (%) | 97.2 93-98 | 97.3 93-99 | 97.2 95-98 | 97.0 93-98 | 97.1 96-98 | 97.1 95-98 | 97.2 95-98 | 97.0 94-98 | 97.2 95-98 | 97.0 94-98 | 96.9 95-98 | 96.8 94-98 | 97.1 95-98 | 96.3 94-98 | 96.4 94-98 | 96.1 93-97 | 97.5 96-98 | 97.4 97-99 | 97.3 97-99 | 97.3 96-99 |
| Hemodynamic parameters | | | | | | | | | | | | | | | | | | | | |
| HR (min^-1^) | 84  54-113 | 82  66-107 | 82  55-112 | 85  69-108 | 102  75-134 | 101  78-131 | 101  78-129 | 104  79-130 | 117  84-148 | 120  88-157 | 116  90-150 | 121  84-155 | 139  96-166 | 141  103-173 | 142  96-171 | 145  102-179 | 108  78-167 | 112  73-163 | 115  76-172 | 117  74-179 |
| SBP (mmHg) | 111 86-141 | 114 82-142 | 111 68-149 | 109 94-140 | 120 86-159 | 123 91-169 | 121 88-162 | 121 85-151 | 139 101-180 | 141 109-202 | 140 100-196 | 143 111-197 | 157 108-220 | 159 120-228 | 163 109-233 | 164 91-240 | 135 91-197 | 140 88-203 | 140 84-205 | 142 85-220 |
| DBP (mmHg) | 78 53-108 | 78 56-99 | 79 51-103 | 78 44-104 | 75 51-97 | 76 58-119 | 72 54-111 | 70 49-102 | 76 58-94 | 78 50-121 | 73 56-114 | 75 58-122 | 75 54-108 | 81 57-104 | 79 53-110 | 76 50-129 | 75 55-105 | 74 50-94 | 77 41-100 | 75 50-101 |
| Capnometric parameters | | | | | | | | | | | | | | | | | | | | |
| pCO_2_in (mmHg) | 4.46 2.9-12 | 6.40 4.3-12 | 6.59 4.5-9.1 | 6.56 4.1-17 | 4.59 3.6-6.1 | 6.88 4.9-13 | 6.37 4.6-11 | 7.69 5.0-15 | 4.56 3.8-6.7 | 6.61 4.8-13 | 6.83 4.9-10 | 9.02 4.9-13 | 4.71 3.6-6.0 | 7.10 4.6-14 | 7.15 4.7-9.5 | 8.98 5.2-17 | 4.47 3.6-9.0 | 6.19 4.3-14 | 6.78 4.5-9.3 | 7.62 4.3-17 |
| pCO_2_ex (mmHg) | 27.01 19-32 | 29.50 22-40 | 29.74 20-36 | 30.22 22-37 | 30.47 24-36 | 32.79 25-42 | 32.58 23-44 | 33.86 26-39 | 30.73 23-37 | 33.31 23-45 | 32.89 24-42 | 34.50 26-41 | 28.52 20-38 | 32.89 20-46 | 32.13 20-42 | 34.12 23-45 | 27.13 19-32 | 29.51 17-40 | 28.53 17-35 | 29.72 20-35 |
| Mask microclimate | | | | | | | | | | | | | | | | | | | | |
| Tmask (°C) | 29.2 22-31 | 32.3 22-35 | 32.7 28-35 | 34.0 28-35 | 28.7 26-32 | 32.3 31-35 | 32.6 31-34 | 34.1 32-35 | 28.3 25-30 | 31.5 30-34 | 32.1 30-34 | 33.6 32-35 | 28.1 25-30 | 31.2 29-33 | 31.7 29-34 | 33.4 32-35 | 28.2 24-31 | 31.6 29-35 | 32.0 29-35 | 33.6 32-36 |
| RH (%) | 42.4 21-92 | 70.8 23-95 | 77.0 37-100 | 75.0 27-97 | 43.5 17-88 | 72.7 59-97 | 77.2 67-100 | 76.2 59-99 | 44.9 16-89 | 72.7 49-98 | 79.8 67-100 | 75.6 59-100 | 47.2 16-85 | 74.5 52-94 | 81.4 68-100 | 79.1 62-100 | 48.0 19-31 | 78.0 60-100 | 84.5 69-100 | 81.3 68-100 |

BORG: Borg scale; CM: community mask;; DBP: Diastolic blood pressure; EELV: FFP2: filtering face piece class 2; HR: Heart rate; NM: no mask; pCO_2_: partial pressure of carbon dioxide; pO_2_: partial pressure of oxygen; pCO_2_ex: expiratory carbon dioxide pressure; pCO_2_in: inspiratory carbon dioxide pressure, pH: potential of hydrogen in blood; RH: relative humidity; RR: Respiratory rate; SBP: Systolic blood pressure; sO_2_: haemoglobin oxygen saturation; SM: surgical mask; Tmask: temperature behind the mask; Ttot: inspiratory and expiratory time.

Table S6: Correlations of body plethysmography and ergometry results (Pearson Correlation (r) and 95% CI with Fisher transformation))

| NM | | Ergometry parameters | | | | | | | | | | |
| --- | --- | --- | --- | --- | --- | --- | --- | --- | --- | --- | --- | --- |
| Body plethysmography parameter |  | BORG | | pCO_2_in | | Ttot | | pCO_2_ | | pO_2_ | | sO_2_ |
|  | FVC | 0.005  (-0.134;0.144) | | 0.224  (0.087;0.351) | | 0.11  (-0.03;0.24) | | 0.207  (0.070;0.336) | | 0.089  (-0.051;0.224) | | 0.195  (0.057;0.324) |
|  | FEV_1_ | -0.006  (-0.145;0.133) | | 0.210  (0.073;0.338) | | 0.05  (-0.09;0.18) | | 0.267  (0.133;0.391) | | 0.083  (-0.056;0.219) | | 0.186  (0.048;0.316) |
|  | Rtot | -0.037  (-0.175;0.102) | | -0.067  (-0.203;0.073) | | -0.03  (-0.17;0.11) | | -0.157  (-0.290;-0.019) | | -0.147  (-0.280;-0.009) | | -0.212  (-0.340;-0.075) |
|  | sRtot | -0.020  (-0.158;0.120) | | -0.021  (-0.159;0.118) | | 0.15  (0.01;0.29) | | -0.057  (-0.194;0.083) | | -0.157  (-0.289;-0.018) | | -0.139  (-0.273;-0.000) |
|  | WOB | -0.016  (-0.154;0.123) | | 0.097  (-0.042;0.233) | | 0.21  (0.07;0.34) | | 0.030  (-0.109;0.168) | | 0.011  (-0.128;0.149) | | -0.040  (-0.178;0.099) |
|  | RP | -0.020  (-0.158;0.119) | | 0.081  (-0.058;0.218) | | 0.15  (0.01;0.28) | | 0.011  (-0.128;0.150) | | -0.046  (-0.183;0.094) | | -0.102  (-0.237;0.038) |
|  | Ttot | 0.059  (-0.080;0.196) | | 0.007  (-0.132;0.145) | | 0.19  (0.05;0.32) | | 0.047  (-0.093;0.184) | | 0.091  (-0.049;0.226) | | 0.129  (-0.010;0.263) |
| SM | | Ergometry parameters | | | | | | | | | | |
| Body plethysmography parameter | FVC | -0.02  (0.16;0.11) | -0.05  (-0.18;0.09) | | 0.16  (0.02;0.29) | | 0.10  (-0.04;0.23) | | 0.15  (0.01;0.28) | | 0.25  (0.12;0.38) | |
|  | FEV_1_ | -0.03  (-0.16;0.11) | -0.03  (-0.16;0.11) | | 0.10  (-0.04;0.23) | | 0.16  (0.02;0.29) | | 0.18  (0.04;0.31) | | 0.25  (0.11;0.37) | |
|  | Rtot | -0.01  (-0.15;0.13) | -0.08  (-0.22;0.06) | | -0.01  (-0.15;0.13) | | -0.28  (-0.41;-015) | | 0.16  (0.02;0.29) | | 0.07  (-0.07;0.21) | |
|  | sRtot | -0.02  (-0.16;0.11) | -0.13  (-0.26;0.01) | | 0.17  (0.03;0.30) | | -0.17  (-0.31;-0.04) | | 0.15  (0.01;0.28) | | 0.13  (-0.01;0.27) | |
|  | WOB | -0.02  (-0.15;0.12) | -0.03  (-0.17;0.11) | | 0.11  (-0.03;0.24) | | 0.01  (-0.13;0.15) | | 0.02  (-0.12;0.16) | | -0.00  (-0.14;0.14) | |
|  | RP | -0.02  (-0.16;0.12) | -0.03  (-0.17;0.11) | | 0.05  (-0.09;0.18) | | -0.05  (-0.19;0.09) | | 0.06  (-0.08;0.20) | | 0.04  (-0.10;0.18) | |
|  | Ttot | 0.02 (-0.12;0.16) | 0.01  (-0.13;0.14) | | 0.13  (-0.01;0.26) | | 0.22  (0.09;0.35) | | -0.11  (-0.24;0.03) | | -0.06  (-0.20;0.08) | |
| CM | | Ergometry parameters | | | | | | | | | | |
| Body plethysmography parameter | FVC | -0.04  (-0.17;0.10) | -0.02  (-0.16;0.12) | | 0.14  (-0.00;0.27) | | 0.06  (-0.08;0.20) | | 0.08  (-0.06;0.22) | | 0.22  (0.08;0.34) | |
|  | FEV_1_ | -0.05  (-0.19;0.09) | 0.04  (-0.10;0.18) | | 0.06  (-0.08;0.20) | | 0.10  (-0.04;0.24) | | 0.10  (-0.04;0.23) | | 0.21  (0.07;0.34) | |
|  | Rtot | -0.00  (-0.14;0.14) | 0.04  (-0.10;0.18) | | -0.06  (0.20;0.08) | | -0.12  (-0.25;0.02) | | 0.06  (-0.08;0.20) | | -0.05  (-0.18;0.09) | |
|  | sRtot | -0.02  (-0.16;0.12) | -0.03  (-0.17;0.10) | | 0.17  (0.03;0.30) | | 0.00  (-0.14;0.14) | | 0.10  (-0.04;0.23) | | 0.01  (-0.13;0.15) | |
|  | WOB | -0.07  (-0.21;0.07) | -0.01  (-0.15;0.13) | | 0.11  (-0.03;0.25) | | -0.01  (-0.15;0.13) | | 0.21  (0.07;0.34) | | 0.13  (-0.01;0.27) | |
|  | RP | -0.05  (-0.19;0.09) | -0.01  (-0.15;0.13) | | 0.19  (0.05;0.32) | | 0.05  (-0.09;0.19) | | 0.17  (0.03;0.30) | | 0.07  (-0.07;0.21) | |
|  | Ttot | -0.01  (-0.15;0.13) | 0.13  (-0.01;0.27) | | 0.20  (0.07;0.33) | | 0.21  (0.07;0.34) | | -0.14  (-0.28;-0.01) | | -0.07  (-0.21;0.07) | |
| FFP2 | | Ergometry parameters | | | | | | | | | | |
| Body plethysmography parameter | FVC | -0.08  (-0.22;0.06) | -0.21  (-0.34;-0.07) | | 0.19 (0.05;  0.32) | | 0.10  (-0.04;0.23) | | 0.25  (0.11;0.37) | | 0.31  (0.18;0.43) | |
|  | FEV_1_ | -0.08  (-0.22;0.06) | -0.18  (-0.32;-0.04) | | 0.11  (-0.03;0.25) | | 0.13  (-0.01;0.27) | | 0.28  (0.15;0.41) | | 0.32  (0.19;0.44) | |
|  | Rtot | -0.00  (-0.14;0.14) | 0.06  (-0.08;0.20) | | 0.03  (-0.11;0.17) | | -0.06  (-0.19;0.08) | | -0.09  (-0.23;0.05) | | -0.10  (-0.24;0.04) | |
|  | sRtot | -0.01  (-0.15;0.13) | -0.00  (-0.14;0.14) | | 0.20  (0.06;0.33) | | 0.00  (-0.13;0.14) | | -0.02  (-0.16;0.11) | | -0.02  (-0.15;0.12) | |
|  | WOB | -0.08  (-0.22;0.07) | -0.03  (-0.17;0.11) | | 0.17  (0.03;0.31) | | -0.05  (-0.19;0.09) | | 0.10  (-0.04;0.23) | | 0.12  (-0.02;0.25) | |
|  | RP | -0.07  (-0.21;0.07) | -0.11  (-0.25;0.03) | | 0.19  (0.06;0.33) | | -0.05  (-0.19;0.09) | | 0.15  (0.01;0.28) | | 0.12  (-0.02;0.25) | |
|  | Ttot | 0.01  (-0.13;0.15) | -0.04  (-0.18;0.10) | | 0.11  (-0.03;0.24) | | 0.26  (0.13;0.39) | | -0.23  (-0.36;-0.10) | | -0.20  (-0.33;-0.07) | |

BORG: Borg scale; CM: community mask; FEV_1_: forced expiratory volume in 1 s; FFP2: filtering face piece class 2; FVC: forced vital capacity; NM: no mask; RP: respiratory power; Rtot: total airway resistance; WOB: work of breathing; pCO_2_in: inspiratory carbon dioxide pressure; pCO_2_: partial pressure of carbon dioxide; pO_2_: partial pressure of oxygen; SM: surgical mask; sO_2_: haemoglobin oxygen saturation; sRtot: specific airway resistance; Ttot: inspiratory and expiratory time.
